# Reconfigurable Micro/Nano‐Optical Devices Based on Phase Transitions: From Materials, Mechanisms to Applications

**DOI:** 10.1002/advs.202306344

**Published:** 2024-03-15

**Authors:** Chensheng Li, Ruhao Pan, Changzhi Gu, Haiming Guo, Junjie Li

**Affiliations:** ^1^ Beijing National Laboratory for Condensed Matter Physics Institute of Physics Chinese Academy of Sciences Beijing 100190 China; ^2^ CAS Key Laboratory of Vacuum Physics School of Physical Sciences University of Chinese Academy of Sciences Beijing 100049 China; ^3^ Songshan Lake Materials Laboratory Dongguan Guangdong 523808 China

**Keywords:** integrated photonic systems, micro/nano‐optical devices, phase change materials, reconfigurable, tunable optical responses

## Abstract

In recent years, numerous efforts have been devoted to exploring innovative micro/nano‐optical devices (MNODs) with reconfigurable functionality, which is highly significant because of the progressively increasing requirements for next‐generation photonic systems. Fortunately, phase change materials (PCMs) provide an extremely competitive pathway to achieve this goal. The phase transitions induce significant changes to materials in optical, electrical properties or shapes, triggering great research interests in applying PCMs to reconfigurable micro/nano‐optical devices (RMNODs). More specifically, the PCMs‐based RMNODs can interact with incident light in on‐demand or adaptive manners and thus realize unique functions. In this review, RMNODs based on phase transitions are systematically summarized and comprehensively overviewed from materials, phase change mechanisms to applications. The reconfigurable optical devices consisting of three kinds of typical PCMs are emphatically introduced, including chalcogenides, transition metal oxides, and shape memory alloys, highlighting the reversible state switch and dramatic contrast of optical responses along with designated utilities generated by phase transition. Finally, a comprehensive summary of the whole content is given, discussing the challenge and outlooking the potential development of the PCMs‐based RMNODs in the future.

## Graphical abstract

Reconfigurable micro/nano‐optical devices (RMNODs) are particularly significant for their optical responses can be reversibly modulated, further enabling unprecedented opportunities for next‐generation photonic systems. This review systematically summarizes the RMNODs based on phase transitions, spanning from materials and phase change mechanisms to applications. The RMNODs consisting of chalcogenides, transition metal oxides, and shape memory alloys are emphatically introduced.

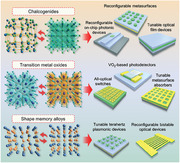

## 1 Introduction

Optical devices with micro/nano‐structures have capabilities to emit, guide, modulate, localize, absorb, and detect light at sub‐wavelength scales. According to the mechanisms and optical characteristics, these devices are mainly categorized as nanolasers,^[^ ^1^, ^2^, ^3^, ^4^, ^5^ ^]^ metasurfaces,^[^ ^6^, ^7^, ^8^, ^9^, ^10^ ^]^ optoelectronic devices,^[^ ^11^, ^12^, ^13^, ^14^, ^15^ ^]^ optical waveguides,^[^ ^16^, ^17^, ^18^, ^19^, ^20^ ^]^ optical films,^[^ ^21^, ^22^ ^]^ microcavities,^[^ ^23^, ^24^, ^25^ ^]^ photonic crystals,^[^ ^26^, ^27^ ^]^ etc. The development of micro/nano‐optical devices (MNODs) has greatly promoted the progression of photonics systems, which are especially desirable for applications in optical neural networks,^[^ ^28^ ^]^ optical computing,^[^ ^29^, ^30^ ^]^ integrated photonics circuits,^[^ ^31^ ^]^ quantum technologies,^[^ ^32^, ^33^ ^]^ and more. So far, significant achievements have been made in the field of photonic systems which develop as a separate branch in parallel with the evolution of electronic counterpart.^[^ ^34^, ^35^, ^36^ ^]^ Notably, the feature sizes of electronic devices have nearly approached the quantum limit, leading to unprecedented integration and excellent calculation performance.^[^ ^37^ ^]^ While the heating problem and short channel effect are inevitable and cause inferiority.^[^ ^38^ ^]^ Photonic systems, with broader bandwidth and avoidance of signal crosstalk, play indispensable roles in the transmission, memory, processing, and programming of optical signals and are more hopeful of achieving novel technologies. On the other hand, their relatively low level of integration density, operation stability, and conversion efficiency as well as high construction cost remain to be solved. Undoubtedly, the development of prospective photonic systems largely depends on the function improvement of constituent MNODs. It is noteworthy that flexible dynamic modulations are more preferable in active processing of optical signals. While conventional MNODs have limited light field manipulation abilities due to fixed optical responses. Accordingly, the reconfigurable micro/nano‐optical devices (RMNODs) are particularly significant and popular for their optical responses can be reversibly modulated, further enabling unprecedented opportunities for next‐generation photonic systems with high levels of integration, responsiveness, endurance, and efficiency.^[^ ^39^ ^]^ Thus, exploring innovative RMNODs to build photonic systems with distinguished performance is of great realistic necessity.

Up to now, liquid crystals,^[^ ^40^, ^41^, ^42^, ^43^ ^]^ flexible dielectrics,^[^ ^44^ ^]^ nano‐kirigami,^[^ ^45^ ^]^ indium tin oxide (ITO),^[^ ^46^, ^47^ ^]^ semiconductors,^[^ ^48^, ^49^, ^50^ ^]^ graphene,^[^ ^51^, ^52^, ^53^, ^54^ ^]^ etc. have been proven to realize RMNODs under electrical, optical, and stretchable mechanical control, obtaining some meaningful results. However, some challenges remain: for example, the millisecond‐level responding time of liquid crystal makes it unsuitable for ultrafast optical signal processing, mechanical control of flexible dielectric suffers from a slower response and large bulk problems, reconfigurable kirigami cannot tolerate repeated shape change because of metal fatigue. Especially, phase change materials (PCMs) provide another extremely promising strategy that avoids the issues above and accomplishes the aim, which takes advantage of the intrinsic reversibility of its physical state to gain dynamic modulation.^[^ ^55^, ^56^ ^]^ PCMs are characterized by phase transformation in response to certain external stimulation, such as electrical pulse,^[^ ^57^, ^58^ ^]^ laser irradiation,^[^ ^59^, ^60^, ^61^ ^]^ and thermal excitation.^[^ ^62^ ^]^ In particular, Chalcogenide PCMs possess phase transition attributes that switch between amorphous and crystalline states.^[^ ^63^, ^64^ ^]^ GeSbTe (GST) is one of the most well‐established chalcogenide PCMs with non‐volatility, which has been widely used in optical storage media.^[^ ^65^, ^66^ ^]^ Its unity‐order changes in the permittivity across a broad spectrum, robust switching over 10^9^ cycles, and fast configuration time (≈100 ns) provide a great convenience for RMNODs to durably fulfill fast modulation within broadband.^[^ ^63^, ^67^ ^]^ Moreover, VO_2_, a most studied transition metal oxide, is also a high‐profile PCM that features dramatic contrasts in permittivity as well as electrical conductivity owing to the metal‐insulator transition (MIT) coupled with a structural phase transition.^[^ ^68^, ^69^, ^70^ ^]^ Being different from chalcogenide PCMs, VO_2_ is a volatile material, which means the optical devices based on VO_2_ could interact with the light wave in a self‐adaptive manner. The extremely short phase transformation response time (≈5 ps) makes VO_2_ a prominent material in ultrafast reconfigurable photonic applications.^[^ ^71^ ^]^ Furthermore, shape memory alloys (SMAs) are another important kind of PCMs, which have the ability to return to a former shape when subjected to an appropriate activation, exhibiting peculiar thermomechanical behaviors under mechanical and thermal conditions.^[^ ^72^, ^73^, ^74^ ^]^ The shape reconfiguration processes can reach millions of times. Among SMAs materials, NiTi is a typical representative due to its unique mechanical performance such as fatigue resistance and pseudoplasticity. The geometry transitions can change the surface electric field and current distributions, which are useful for active plasmonic device implementations. Consequently, the unparalleled virtues, accompanied by the simple compositions and relatively easy synthesis process make these PCMs of intense focus for multifunctional RMNODs.

**Figure 1:**
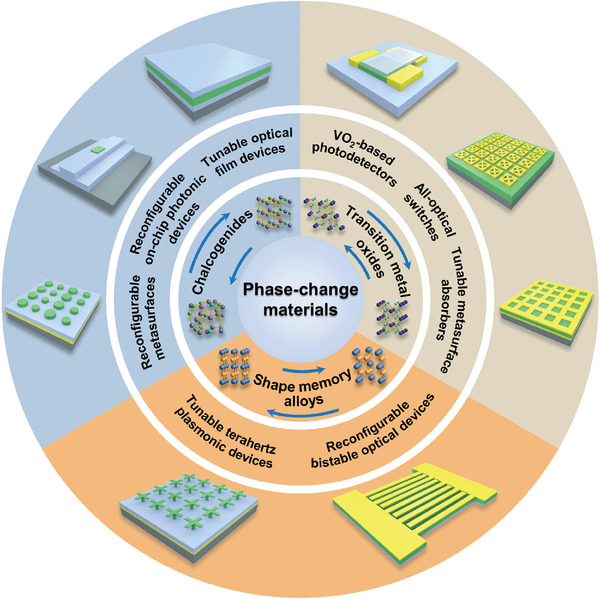
Summary of representative PCMs and their applications in RMNODs. Typical architectures of PCM‐based RMNODs are illustrated in the outer circle of the figure, and the PCMs are depicted in green.

There have existed several review articles to overview the optical research progress and applications of PCMs,^[^ ^39^, ^63^, ^75^, ^76^, ^77^, ^78^, ^79^, ^80^, ^81^ ^]^ which focused mostly on metasurfaces with non‐volatile and tunable electromagnetic functionality. These reviews offer very good references to advanced scientific research and commercial manufacturing. While the basic phase transition mechanism and other reconfigurable optical devices based on PCMs are equally important and should be taken seriously. A review that detailly illustrates the material properties and physical origin of phase transitions of PCMs as well as their applications in RMNODs is constructive to the future development of reconfigurable photonic systems. In this review, we comprehensively summarize PCMs and their recent applications in micro/nano‐optical devices with reversible tunability. Three kinds of representative PCMs (chalcogenides, transition metal oxides, and shape memory alloys) are emphatically introduced, as shown in **Figure** **1** In the following sections, we first illustrate the mechanism of phase change and properties of PCMs, highlighting the multi‐stimuli responsive crystal structure transition and subsequent changes in permittivity, resistance, and shapes. Then, RMNODs comprising these PCMs are in turn introduced. At the end of the paper, we give a summary of the whole content and provide the outlook for the challenge and future development.

## 2 Mechanism of Phase Change and Properties of PCMs

**Figure 2:**
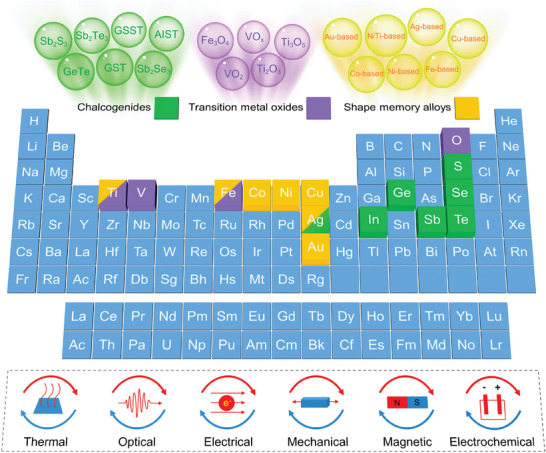
Element components of chalcogenides, transition metal oxides, and shape memory alloys with phase change attributes and the excitation methods for phase transition.

**Figure 3:**
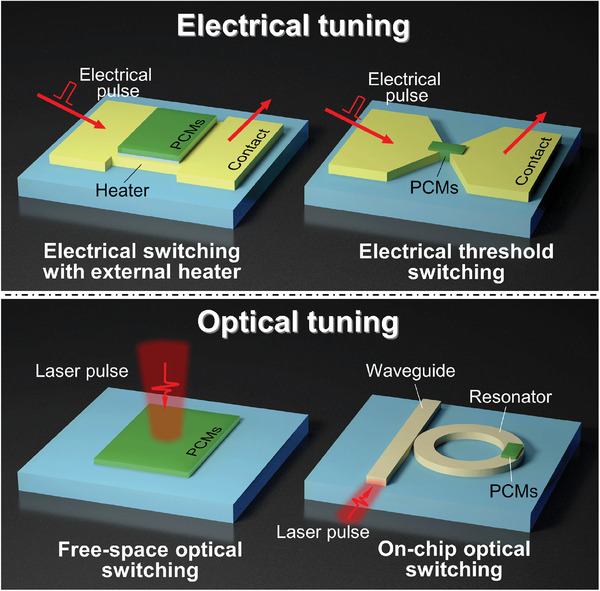
Schematic of electrical and optical tuning schemes for stimulating phase transitions of PCMs in RMONDs. The PCMs in the devices are exhibited in green.

Phase transitions describe the process that the crystal or electronic structure of a uniform material system transforms to a new physical state with order parameters and symmetry distinct from the original ones.^[^ ^82^ ^]^ There are various material systems with phase transition properties, such as chalcogenides (e.g., GST, Ge_2_Sb_2_Se_4_Te (GSST), AgInSbTe (AIST), GeTe, Sb_2_S_3_, Sb_2_Se_3_, and Sb_2_Te_3_),^[^ ^63^, ^66^, ^83^, ^84^ ^]^ transition metal oxides (e.g., VO_2_, VO_x_ (x≠2), Fe_3_O_4_, Ti_2_O_3_, and Ti_3_O_5_),^[^ ^68^, ^85^ ^]^ shape memory alloys (e.g., NiTi‐based, Fe‐based, Cu‐based, Co‐based, Ni‐based, Ag‐based, and Au‐based alloys),^[^ ^72^, ^86^, ^87^ ^]^ and superconductors^[^ ^88^, ^89^, ^90^ ^]^ (e.g., YBaCuO, LaO_1‐x_F_x_FeAs, Ba_1‐x_K_x_Fe_2_As_2_, and MgB_2_). In particular, chalcogenides, transition metal oxides, and shape memory alloys are the main focuses of this review, whose element components are shown in **Figure** **2** Various excitation methods can be adopted to induce phase transitions, including thermal, optical, electrical, mechanical, magnetic, and electrochemical stimulations.^[^ ^78^, ^91^ ^]^ Among them, electrical and optical tuning are most valuable due to the realization of high integration, high reliability, and ultrafast modulation, as shown in **Figure** **3** In detail, the electrical tuning schemes can be classified as electrical switching with external heater and electrical threshold switching.^[^ ^58^, ^92^ ^]^ For the former way, different kinds of materials can be used to fabricate external heaters (e.g., Ag,^[^ ^57^ ^]^ Au,^[^ ^93^ ^]^ Ti/Pt,^[^ ^58^ ^]^ W,^[^ ^94^ ^]^ ITO,^[^ ^95^, ^96^, ^97^ ^]^ doped Si,^[^ ^98^, ^99^ ^]^ PIN diode,^[^ ^100^, ^101^ ^]^ and graphene^[^ ^102^ ^]^). Usually, Al_2_O_3_, with relatively high thermal conductance in dielectrics, is deposited between PCMs and metal heaters as a diffusion barrier to prevent their direct contact.^[^ ^57^, ^58^, ^93^ ^]^ Besides, SiO_2_, Si_3_N_4_, and ITO are other alternative dielectric material.^[^ ^103^, ^104^, ^105^ ^]^ On the other hand, the optical tuning schemes can be classified as free‐space optical and on‐chip optical switching.^[^ ^60^, ^106^ ^]^ Both electrical and optical schemes usually adopt a conformal dielectric film as a protection layer, where Al_2_O_3_ is the most common choice,^[^ ^57^, ^58^, ^93^, ^100^ ^]^ and Si_3_N_4_,^[^ ^103^, ^105^, ^107^ ^]^ SiO_2_,^[^ ^96^, ^108^ ^]^ and ITO^[^ ^98^, ^106^ ^]^ can also be adopted.

**Figure 4:**
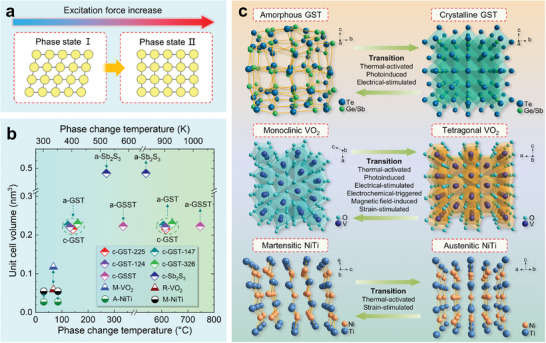
Structure phase transitions of the representative PCMs. a) Structure phase transition from phase state I to phase state II stimulated by external excitation force. b) Phase change temperatures of the PCMs. c) Crystal structure diagrams and the driving forces of phase change for GST, VO_2_, and NiTi.

According to the derivative variations for thermodynamic potentials at the critical point, phase transitions can be divided into first‐order transition (e.g., structure transition, solid‐liquid transition), second‐order transition (e.g., superconducting transition, ferromagnetic transition, and antiferromagnetic transition), and higher‐order phase change (e.g., Bose‐Einstein condensate).^[^ ^109^ ^]^ Structure transition is one of the most common types. With excitation forces increasing, structure transition occurs when the new phase of the matter is more thermodynamically stable than the initial state (**Figure** **4**a).^[^ ^109^, ^110^ ^]^ The concerned PCMs (chalcogenides, transition metal oxides, and SMAs) have intrinsic structural phase transition attributes, which dominate the reconfigurable properties and radical optical contrasts of RMNODs. Among them, GST, VO_2_, and NiTi alloy are the typical representatives, whose crystal structures, phase transition driving forces, physical properties, and property contrast origins are detailly presented. Some other chalcogenides PCMs (e.g., AIST, Sb_2_S_3_, GSST, and GeTe) and shape memory alloys (e.g., NiTiCu), which share similar properties and same phase transition mechanism with the three representative materials, will be included in the application section.

First, the crystal structures of the representative PCMs and the excitation methods for their structure transitions are comprehensively introduced. All the phase transition behaviors discussed here are at ambient pressure, which is reasonable for the accordance with the regular operation atmosphere of photonic systems. The phase transition temperatures and corresponding unit cell volumes are summarized and shown in Figure 4b.

GST is one of the well‐established PCMs which has been widely used as data storage media.^[^ ^66^, ^67^ ^]^ Beyond that, it is being reborn in the area of micro/nano‐optics as a key constituent part of tunable optical devices.^[^ ^59^ ^]^ GST alloys with disparate stoichiometric ratios originate from GeTe‐Sb_2_Te_3_ pseudobinary alloy system.^[^ ^83^, ^111^ ^]^ GeTe possesses a high optical contrast between amorphous and crystalline states but a slow crystallization rate. To make up for this deficiency, Sb_2_Te_3_ which crystallizes rapidly is introduced to comprise the ternary alloy with both excellent features. The most common and valuable sorts are Ge_2_Sb_2_Te_5_ (GST‐225), Ge_3_Sb_2_Te_6_ (GST‐326), Ge_1_Sb_4_Te_7_ (GST‐147), and Ge_1_Sb_2_Te_4_ (GST‐124). Moreover, by substituting a part of Te atoms with selenium in GST‐225, a composition‐optimized PCM GSST which belongs to GST family is developed.^[^ ^108^, ^112^, ^113^ ^]^ With the temperature increasing, amorphous GST (a‐GST) first crystallizes into face‐centered cubic (FCC) phase (c‐GST) with space group $Fm\bar{3}m$ (No. 225) at glass transition temperature (crystallization temperature) *T_g_* of ≈140 °C (Figure 4b). The *T_g_* monotonically increases with increasing GeTe content.^[^ ^111^ ^]^ The lattice parameters and unit cell volumes of c‐GST would have tiny variations for different stoichiometric ratios.^[^ ^114^ ^]^ At a higher temperature of ≈300 °C, the c‐GST transforms to the hexagonal structure. After quenching over melting temperature *T_m_* of ≈600 °C, the crystalline state returns to amorphousness.^[^ ^111^, ^114^ ^]^ In this review, we focus on the transition between amorphous and FCC states. As clearly illustrated in Figure 4c, the crystal structure of a‐GST is disordered and without symmetry. After the phase transition, the structure of c‐GST shows long‐range order. In this case, c‐GST possesses a distorted rock‐salt‐like structure with an ordered Te sublattice occupying the 4a Wyckoff position and a disordered sublattice of Ge/Sb/vacancies occupying the 4b Wyckoff position.^[^ ^115^, ^116^ ^]^ The Ge and Sb atoms in the crystal lattice are displaced off‐center and the distorted octahedral atomic arrangements of Ge and Sb atoms exist.^[^ ^116^, ^117^ ^]^ The phase transformation of GST is thermal‐activated. More detailly, the excitation methods include applying annealing/quenching process,^[^ ^62^, ^118^ ^]^ electrical pulse,^[^ ^57^, ^58^ ^]^ and laser pulse,^[^ ^59^, ^60^ ^]^ which are essentially dependent on the heating effect.

As a strongly correlated electron material, VO_2_ has triggered great interest in scientific research for its well‐known MIT accompanied by structure phase change.^[^ ^119^ ^]^ Below critical temperature *T_MIT_* of ≈68 °C, VO_2_ adopts a monoclinic (M‐VO_2_) structure belonging to space group *P2_1_/c* (No. 14) with unit cell parameters a ≈ 5.75 Å, b ≈ 4.53 Å, c ≈ 5.38 Å, and β ≈ 122.6°.^[^ ^68^, ^119^, ^120^, ^121^ ^]^ At high temperatures above *T_MIT_*, VO_2_ crystallizes into tetragonal rutile (R‐VO_2_) with space group *P4_2_/mmm* (No. 136) and unit cell parameters a = b ≈ 4.55 Å and c ≈ 2.85 Å.^[^ ^68^, ^120^ ^]^ Additional phases, triclinic, space group $P\bar{1}$ (No. 2) and monoclinic, space group C2/m (No. 12), are sub‐stable states, which can be stabilized by applying strain or doping with lower‐valence metal atoms such as Al, Cr, Ga, and Fe.^[^ ^68^, ^122^, ^123^, ^124^, ^125^ ^]^ As shown in Figure 4c, for both the M and R phases, each vanadium atom is surrounded by an octahedral arrangement of oxygen atoms. While the octahedral coordination geometries remain irregular due to the unequal band lengths of V‐O, exhibiting Jahn‐Teller distortion and much severer distortion in the R and M phases, respectively.^[^ ^126^ ^]^ The R‐VO_2_ crystal structure is characterized by linearly aligned edge‐sharing VO_6_ octahedra along the c‐axis with unique V‐V intervals of ≈2.85 Å. For the M‐VO_2_, the VO_6_ octahedra are no longer quite on a straight line for its tilting, displaying a zigzag chain with alternating V‐V distances of 2.65 Å and 3.12 Å along the a‐axis.^[^ ^126^, ^127^, ^128^ ^]^ VO_2_ is a multi‐stimuli responsive material, meaning that its crystallographic transition can be induced by diverse excitations including thermal,^[^ ^129^ ^]^ optical,^[^ ^130^ ^]^ electrical,^[^ ^102^, ^131^ ^]^ electrochemical,^[^ ^132^ ^]^ magnetic,^[^ ^133^ ^]^ and mechanical stimuli.^[^ ^134^ ^]^

SMAs are a magical class of alloys that can recover their original shape by heating after a large deformation.^[^ ^135^ ^]^ The resonance modes of the SMAs‐based MNODs can be adjusted by reversibly switching the structure between martensitic and austenitic phases, thus leading to tunable optical responses. NiTi‐based SMAs contain NiTi, NiTiCu, NiTiFe, NiTiPd, NiTiNb, NiTiCo, etc.^[^ ^72^ ^]^ In actual applications, the most common material is NiTi, which has been widely used in biomedical, aerospace, robotics, and automotive fields. As displayed in Figure 4b,c, the martensite phase of NiTi (M‐NiTi) is stable at a low‐temperature level with a monoclinic structure (space group *P2_1_/m* (No. 11)) and lattice parameters a ≈ 2.898 Å, b ≈ 4.108 Å, c ≈ 4.646 Å, and β ≈ 97.78°.^[^ ^136^ ^]^ The NiTi austenite phase (A‐NiTi) remains stable at a high‐temperature level with a cubic structure (space group $Pm\bar{3}m$ (No. 221)) and lattice parameters a = b = c ≈ 3.011Å.^[^ ^137^ ^]^ Upon cooling through the martensitic phase transformation, the unit cell volume is doubled in size due to the loss of partial symmetry arising from the displacement of atoms. The phase transition of NiTi can be thermal‐activated and strain‐stimulated.^[^ ^74^ ^]^ Normally, the transitions are excited by direct heating and cooling.

The above introduction of the crystal structures and the excitation methods for state transition provides a clear and intuitionistic perspective to comprehend the representative PCMs. Then, we will discuss in detail the physical origin of contrasts induced by phase transitions of the representative PCMs and their physical properties in the following sections.

**Figure 5:**
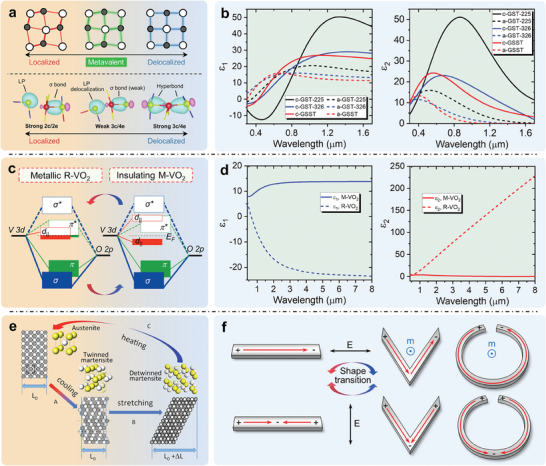
Physical origins of property contrasts induced by phase transitions of the representative PCMs and their physical properties. a) Metavalent (top) and Hyperbonding (bottom) models to explain the phase change behavior of GST. Reproduced with permission.^[^ ^138^ ^]^ Copyright 2018, Wiley‐VCH. Reproduced with permission.^[^ ^139^ ^]^ Copyright 2020, Wiley‐VCH. b) Wavelength‐dependent permittivity of GST and GSST. Reproduced with permission.^[^ ^112^ ^]^ Copyright 2018, Optica Publishing Group. Reproduced with permission.^[^ ^57^ ^]^ Copyright 2021, Springer Nature. c) Energy band structures of metallic R‐VO_2_ and insulating M‐VO_2_. Reproduced with permission.^[^ ^141^ ^]^ Copyright 2013, Nature Publishing Group. d) Wavelength‐dependent permittivity of VO_2_. Reproduced under the terms of the CC‐BY Creative Commons Attribution 4.0 International license.^[^ ^146^ ^]^ Copyright 2009, Optical Society of America. e) Schematic of reversible phase transition of NiTi alloy. Reproduced under the terms of the CC‐BY Creative Commons Attribution 3.0 Unported license.^[^ ^144^ ^]^ Copyright 2016, The authors, published by Elsevier. f) Charge distributions of the metallic nanorods antenna, V‐shaped antennas, and SRR under electrical fields with different polarization directions. Reproduced with permission.^[^ ^145^ ^]^ Copyright 2014, Nature Publishing Group.

The mainstream view believes that the coordination numbers of atoms in a‐GST are generally in agreement with the 8‐N rule of covalent bonding, whereas in c‐GST, pronounced electron delocalization results in a significant increase of electronic polarizability, which is a characteristic of resonance bonding.^[^ ^63^, ^84^ ^]^ Consequently, the great differences in permittivity between c‐GST and a‐GST are attributed to the drastic change in their chemical bonding. The permittivity of a‐GST is that expected of a covalent semiconductor, and that of c‐GST is strongly enhanced by resonant bonding effects. However, recent research supports the idea that GST has a unique bonding mode that is fundamentally different from resonant bonding. Wuttig et al. suggested that the nature of bonding within c‐GST is between the localized covalency and the delocalized metallicity, as shown in the top part of **Figure** **5**a, which is called metavalent.^[^ ^138^ ^]^ Afterwards, Lee et al. proposed a new concept of multicenter hyperbonding.^[^ ^139^ ^]^ It is suggested that there are two strong bonding interactions, ordinary two‐center/two‐electron (2c/2e) and three‐center/four‐electron (3c/4e) hyperbond, and a weak 3c/4e interaction for the bonding in GST (bottom part of Figure 5a). Due to the confinement of lattice symmetry, at most three perpendicular 3c/4e hyperbonds would form around the Ge/Sb atoms, causing a higher proportion of hyperbonds in c‐GST than that in a‐GST. Although the two views are different, both of them support the idea that more delocalized bonding‐electrons exist in c‐GST, which is responsible for the larger permittivity (both in real and imaginary parts) compared with a‐GST (Figure 5b). Besides, the optical performance of GST can be controlled by tuning the GeTe:Sb_2_Te_3_ ratio.

The phase transition generates substantial changes in the optical and electrical properties of VO_2_. To understand the physical origin behind, the electronic band structures of the two distinct phases of VO_2_ are presented in Figure 5c. For R‐VO_2_, the density of states at the Fermi level (*E_F_*) is determined by the mixture of *d_||_* and π* orbitals.^[^ ^128^, ^140^, ^141^ ^]^ Electrons around *E_F_* dominant the transport processes, which is consistent with that of metals. After the phase transition, the π* orbital elevates and the *d_||_* orbital splits, leading to a bandgap of ≈0.7 eV.^[^ ^140^, ^141^ ^]^ The valence band is full and make no contribution to free carriers. The extreme variation in carrier density triggers great contrast in both permittivity and electrical resistance between R‐VO_2_ and M‐VO_2_. As shown in Figure 5d, in the long wavelength region, the real part of the permittivity of R‐VO_2_ is negative and the imaginary part is extremely large, indicating a typical metallic behavior.^[^ ^93^, ^142^ ^]^ While a distinct trend would be expected for the M‐VO_2_, which accords with dielectrics.^[^ ^93^, ^142^ ^]^ The resistance distinct of VO_2_ induced by phase transition is also obvious.^[^ ^143^ ^]^

The phase transition of NiTi alloy is displacive and diffusionless.^[^ ^137^ ^]^ Due to the shape memory effect, the shapes of NiTi alloy structures can be artificially controlled. The shape memory effects of SMAs originate from thermoelastic martensitic phase transitions.^[^ ^74^ ^]^ Figure 5e illustrates the reversible phase transition processes.^[^ ^144^ ^]^ First, the desired shape is molded at the A‐NiTi state, which can be considered as the programming progress. After cooling down, the austenite transforms to twinned martensite (process A) and the programmed shape remains unchanged. Under external stretching force, twinned martensite would then transform to detwinned martensite (process B) and the shape of NiTi would change. Finally, after heating above the phase change temperature, detwinned martensite changes to austenite state (process C) and the shape recovers to the programmed shape.^[^ ^72^, ^144^ ^]^ The ability of NiTi alloy to memorize a specific shape in the austenite phase is called one‐way shape memory. After deformation training, NiTi can memorize two different shapes in martensite and austenite phases, which is called two‐way shape memory.^[^ ^74^ ^]^ The geometries of micro/nanostructures largely determine the electromagnetic field distribution and thus the resonance modes, further dominate the optical responses of optical devices. An intuitive approach to understanding the influence of shape transitions induced by phase changes is viewing from the perspective of charge carrier separation under the external electric fields. Figure 5f clearly displays the charge distributions of the metallic nanorods antenna as well as its folding (V‐shaped antennas) and bending counterparts (split‐ring resonator (SRR)) under electrical fields with different polarization directions.^[^ ^145^ ^]^ The deformations change the symmetry of the nanorods and the dipolar interactions between its ends, resulting in antisymmetric resonance mode (top) and symmetric resonance mode (bottom) with shifting resonance frequency. One the other hand, utilizing the hysteresis effect and actuation induced by NiTi phase changes can also achieve electromagnetic response modulation.

## 3 Chalcogenides‐Based RMNODs

### 3.1 Reconfigurable Metasurfaces

#### 3.1.1 Electrically Reconfigurable Metasurfaces

Metasurface characterized by artificial sub‐wavelength structures with tailored optical responses has the capacity to manipulate the amplitude, phase, and polarization of the incident light.^[^ ^147^, ^148^, ^149^, ^150^ ^]^ Since its first demonstration using spatially discrete V‐antennas,^[^ ^6^ ^]^ metasurface has been attracting great attention for its unique functions, and it has great potential to substitute conventional bulk optical devices in the future. Metasurface has been evaluated as the most promising technology for the realization of the first generation of practical metamaterial‐based devices.^[^ ^145^ ^]^ Metasurfaces with tunable attributes are more fascinating for the realization of dynamic modulation. Various tunable GST‐based metasurfaces controlled either by electrical pulse or pulsed laser have been proposed.

**Figure 6:**
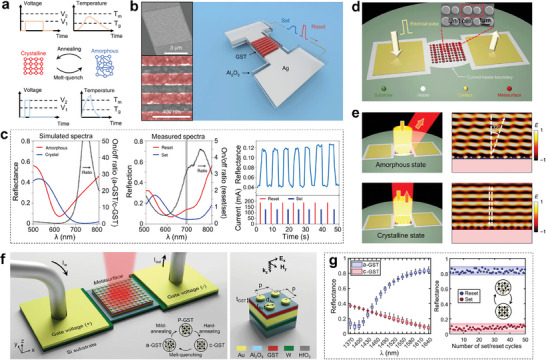
GST‐based metasurfaces with reconfigurability controlled by electrical pulses. a) Electrical pulsing schemes and the temperature profiles of GST nanostructures during (top) crystallization and (bottom) amorphization processes. *T_m_* and *T_g_* denote melting temperature and crystallization temperature, respectively. *V_1_* and *V_2_* denote crystallization voltage and amorphization voltage, respectively. Reproduced with permission.^[^ ^58^ ^]^ Copyright 2021, Springer Nature. b) Schematic and scanning electron micrograph (SEM) images of the electrically tunable metasurface to dynamically control reflected light intensity with GST nano‐grating array. c) Simulated and measured spectra of the reflectance of the metasurface with distinct GST phases. The ratio of the reflectance is shown in the black dotted curve. The right panel is the modulated reflectance signals with reset and set pulses at the gray band of the middle panel. Reproduced with permission.^[^ ^57^ ^]^ Copyright 2021, Springer Nature. d) Schematic of the electrically tunable GSST metasurface with wavefront manipulation functionality, the inset shows the SEM image of the GSST meta‐atoms. e) The simulated electrical field amplitude profiles and the corresponding schematic rendering at 1550 nm for the metasurface with amorphous and crystalline state, respectively. Reproduced with permission.^[^ ^58^ ^]^ Copyright 2021, Springer Nature. f) Schematic illustration of the electrically reconfigurable GST metasurface for the reversible switch of reflectance within broadband in NIR (left) and its cross‐section view (right). The constituent materials are distinguished with different colors. g) Statistical distribution of change in experimentally measured reflectance of the GST metasurface over 50 consecutive cycles for amorphous (blue) and crystalline (red) states for nineteen equally spaced wavelengths (left figure). Cyclability plot of reflectance at 1640 nm during the set (from a‐GST to c‐GST) and reset (from c‐GST to a‐GST) processes (right figure). Reproduced under the terms of the CC‐BY Creative Commons Attribution 4.0 International license.^[^ ^94^ ^]^ Copyright 2022, The authors, published by Springer Nature.

**Figure** **6**a shows the Electrical pulsing schemes and the temperature profiles for the electrothermal effect, where a long duration and low‐voltage (*V_1_)* pulse induces crystallization by heating GST over *T_g_*, and a relatively short and high‐voltage (*V_2_*) pulse triggers amorphization via a fast‐quenching process.^[^ ^58^ ^]^ The electrical controlling of phase transition is conductive to the chip‐scale integrated RMNODs. Wang et al. proposed an electrically tunable phase‐change metasurface containing GST nano‐grating array placed on a silver strip (Figure 6b).^[^ ^57^ ^]^ The reflection of the metasurface varies by tuning the phase state of GST, resulting in a modulation of the reflected light intensity. The highest on/off ratio of 4.5 for the reflectance is achieved at 755 nm. The silver strip is chosen to serve as a mirror to prevent light transmission. Meanwhile, by running through a pulsed current, it can also perform the electrothermal function to achieve a reversible switch between the amorphous and crystalline states of the top GST beams. The simulated and measured spectra of the reflectance are shown in Figure 6c (right and middle), where a single‐band switchable perfect absorption is achieved for the excitation of surface plasmon polaritons (SPPs) on the silver surface and inside the GST beams. The lowest reflection of 4.3% is experimentally demonstrated at ≈700 nm for the crystalline state. After reset by a pulse, the reflection increases to 14.5%, which is proven to be a more than threefold electrical modulation. The reset and set pulses can be implemented repeatedly, and the reflected signal is modulated up and down correspondingly.

Compared with the ternary GST alloys, GSST offers exceptional broadband transparency for both its amorphous and crystalline phase in the NIR region while the large permittivity contrast between the two phases still maintains, which is critical to optical loss reduction.^[^ ^113^ ^]^ Besides, its much larger reversible switching volume brings great convenience to fabricating thick meta‐structure, boosting interaction between light and structures. Wavefront manipulation is an effective way to steer light propagation. An electrically tunable metasurface that can achieve polarization‐independent phase gradience was proposed by Zhang et al. (Figure 6d).^[^ ^58^ ^]^ To obtain an electrothermal uniformity within a large area (up to 0.4×0.4 mm^2^), the boundaries of the Ti/Pt heater are carefully optimized as a parabolic curve to replace the straight geometry in the square heater. Each unit cell of the metasurface contains two GSST cylindrical meta‐atoms. Figure 6e clearly illustrates the realization of beam steering. For the perpendicularly incident light at 1550 nm, the reflected beam deflects at an angle of 32° (+1 order mode) and does not deflect (0 order mode) in the amorphous and crystalline states, respectively. The deflection efficiency for 0 (+1) order mode is measured as 24.8% (8.3%) and the switching contrast ratio is proved to be 8.6 dm.

Another optically reconfigurable GST metasurface tuned by electrical pulse to achieve reversible control of reflectance within a broad NIR band was suggested by Abdollahramezani et al. (Figure 6f).^[^ ^94^ ^]^ Tungsten (W) was chosen as the metallic microheater to drive the phase transition of GST by applying electrical pulses. Both SPPs and localized surface plasmon resonance (LSPR) modes are supported in the GST metasurface. As shown in Figure 6g (left), where the first and third quartiles and the median of the measured data are shown in colored lines, and the maximum/minimum values are represented by black lines, the relatively slight deviation of the measured data under the same state within 50 consecutive cycles reveals the steady and reliable reversible switching process. The resonance wavelength of the metasurface redshifts from 1390 nm for the amorphous state to 1640 nm for the crystalline state, which is attributed to the extinction of SPPs and LSPR modes, respectively. Reflectance changes within 50 cycles at 1640 nm is detailly plotted in Figure 6g (right), where the blue and red shaded areas represent the 95% confidence intervals of ±1% and ±7.5% for amorphous and crystalline states. The average absolute (*ΔR = |R_a‐GST_ – R_c‐GST_|*) and relative (*ΔR/R_c‐GST_*) modulation contrast in reflectance at 1640 nm is over 75% and 1000%, respectively, showing excellent electrical modulation performance.

The above three electrically reconfigurable metasurface designs have experimentally demonstrated the feasibility and multi‐cycle controllability of both amplitude‐type and phase‐type chalcogenide metasurfaces, which offer valuable insights for achieving practical metasurfaces with electrically tunable functionalities in the future. However, due to the introduction of the underneath metal heater, the transmitted light is obstructed and thus only reflective metasurface schemes are accessible. To address this problem, alternative types of external heaters that are transparent to incident light are theoretically proposed to enable transmissive metasurface designs.^[^ ^151^, ^152^, ^153^ ^]^ These schemes are inspiring and remain to be demonstrated through experiments.

#### 3.1.2 Optically Reconfigurable Metasurfaces

**Figure 7:**
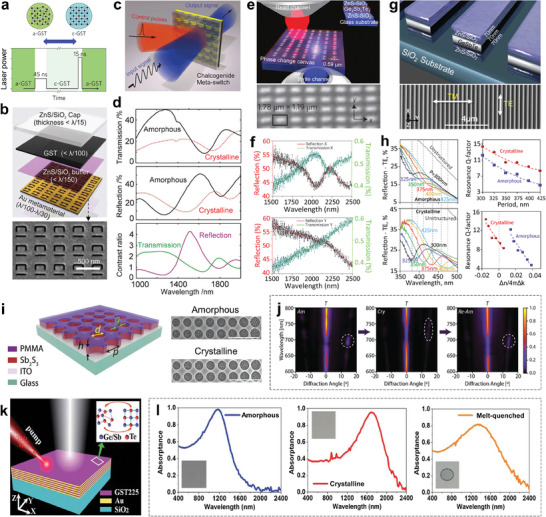
Reconfigurable metasurfaces controlled by pulsed laser. a) Scheme of pulsed laser inducing phase transition of GST. Reproduced with permission.^[^ ^154^ ^]^ Copyright 2016, Springer Nature. b) Multilayer structure of the all‐optical hybrid metasurface and the SEM image of the bottom split ring slots. c) Artistic rendering of the all‐optical metasurface switch. d) The reflection and transmission spectra and the contrast ratio between the amorphous and crystalline states of the switch. Reproduced with permission.^[^ ^59^ ^]^ Copyright 2013, Wiley‐VCH. e) Artistic impression of reconfigurable metasurface directly written on a GST film by laser pulses and the reflection image. f) The reflection and transmission spectra of the reconfigurable metasurface for incident light polarized along the horizontal direction (top) and the vertical direction (bottom). Reproduced with permission.^[^ ^60^ ^]^ Copyright 2016, Springer Nature. g) Schematic of the UV‐HEV dielectric metasurface and the corresponding SEM image. h) The reflection spectra (TE‐polarized incidence) of the dielectric metasurface with amorphous (top) and crystalline (bottom) state of the GST layer. Reproduced with permission.^[^ ^157^ ^]^ Copyright 2019, American Chemical Society. i) Schematic and SEM images of the Sb_2_S_3_ metasurface for wavefront control. The scale bars correspond to 1 µm. j) Angle‐resolved normalized transmission spectra for amorphous (Am), crystallized (Cry), and reamorphized (Re‐Am) metasurfaces. The +1 order diffraction mode is circled with dashed lines. Reproduced with permission.^[^ ^160^ ^]^ Copyright 2022, Wiley‐VCH. k) Schematic illustration of the reconfigurable hyperbolic metamaterial. l) The absorption spectra of the reconfigurable hyperbolic metamaterial with different states of GST layers. The insets in the right, middle, and left panels are the microscope images of the amorphous, crystalline, and reamorphizing hyperbolic metamaterials, respectively. Reproduced under the terms of the Creative Commons Attribution‐Noncommercial 3.0 Unported license.^[^ ^161^ ^]^ Copyright 2021, The authors, published by the Royal Society of Chemistry.

Applying laser pulses is another alternative method to control the reversible phase transition of GST. As displayed in **Figure** **7**a, the laser pulses with low power and long pulse width could trigger phase changing from a‐GST to c‐GST, while short and high‐intensity pulses would generate a momentary quenching process and thus the phase reamorphizing.^[^ ^154^ ^]^ This circumstance is similar to that of applying electrical pulses. The nanosecond laser is the common choice for phase control. Besides, the femtosecond laser can yield the same effect. All‐optical switching allows dynamic reconfiguration of the fiber network, achieving low latency bearing of increased data streams.

Gholipour et al. suggested a non‐volatile all‐optical phase‐change switching scheme by hybridizing GST and nanostructured plasmonic metamaterials (Figure 7b).^[^ ^59^ ^]^ Fano resonance modes are supported by the array of asymmetric split ring slots in the Au film. The all‐optical meta‐switch could modulate the transmitted and the reflected light from NIR to MIR waveband through nanosecond laser control of GST phase state (Figure 7c). The tunable domain is as large as 2000 µm^2^. As shown in Figure 7d, both the transmission and reflection spectra for the amorphous and crystalline states exhibit dissimilarity. After crystallizing, the GST refractive index increases, leading to the resonance red‐shifting. The simultaneous increase in extinction coefficient causes the observed broadening of features and the decrease in average reflection and transmission. Consequently, the contrast ratios at certain wavelengths are obtained, which satisfy the requirement of all‐optical switch modulation.

The reconfigurable universal optical platforms with large freedom degrees are meaningful, on which we can build the desired structure and manipulate the configuration to fulfill the demand. Wang et al. demonstrated a novel reconfigurable metasurface that is directly written on the GST film by femtosecond laser (top panel in Figure 7e).^[^ ^60^ ^]^ The component consists of two layers of ZnS‐SiO_2_ separated by a GST film. Laser‐induced phase transition can be achieved within an extremely small volume of GST, which is down to 0.02 µm^2^. The metasurface comprises an array of rectangular crystalline inclusions in the amorphous GST film (bottom panel in Figure 7e), which is created by pulsed laser writing. The measured reflection and transmission spectra are shown in Figure 7f. For the horizontal (x‐axis) polarization incidence (top panel), the resonant transmission peak and reflection dip at 2040 nm are observed. For the case of vertical (y‐axis) polarization, there exist no resonance signals. This result indicates a primary dielectric metasurface with polarization‐dependent resonance functionality. Further on, the crystalline areas can be erased, namely reamorphized, and rewritten, which is convenient to realize multifunction on the universal component.

The infrared waveband is a preferential region for GST‐based metasurface. There are totally two reasons: The permittivity varies dramatically after GST phase change in the infrared band; The imaginary part of permittivity of GST is relatively low in the infrared region, meaning the low optical loss and the high transparency together with high efficiency. On the other hand, the photonics devices with tunable properties at ultraviolet (UV) and partial high‐energy visible (HEV) bands have valuable applications in sensing, beam deflection, high‐density optical memory, and adaptive optics.^[^ ^155^, ^156^ ^]^ In the UV/HEV spectral region, c‐GST features the optical properties of plasmonic media, and a‐GST features that of dielectric media, and both of them are characterized by low refractive index. A tunable dielectric metasurface with a trilayer nanograting structure is designed to operate light field in the UV‐HEV spectral range (Figure 7g).^[^ ^157^ ^]^ The amorphous to crystalline transition of GST is operated by the femtosecond laser at a wavelength of 730 nm. Reflection resonances with diverse quality‐factor (*Q*) are observed for TE‐polarized (parallel to the grating lines) incident light for both amorphous and crystalline phase states (Figure 7h). The reflection resonance wavelengths are dependent on the period (*P*) of the grating but not the phase of GST. The *Q* (*Q = λ_R_/Δλ*, where *λ_R_* is the reflection resonance wavelength and *Δλ* is the full width at half maximum) decreases with increasing *P*. When the *P* is fixed, *Q* increases when crystallizing. These behaviors of resonance quality can be explained by the balance between the refractive index of GST and ZnS/SiO_2_, which show proportionality with quantity *Δn/4πΔk* ($\Delta n\;=\;n_{GST}\;-\;n_{ZnS/SiO_{2}}$, $\Delta k\;=\;k_{GST}\;-\;k_{ZnS/SiO_{2}}$), as shown in Figure 7h.

GST has a relatively narrow bandgap of ≈0.5–0.7 eV, leading to excessive absorption for optical modulation in the VIS‐NIR region.^[^ ^158^ ^]^ Fortunately, benefitting from its wide bandgap (≈1.7–2 eV), Sb_2_S_3_ has a broad transparency window ranging from 610 nm to the NIR region, which enables optical engineering with low optical loss.^[^ ^158^, ^159^ ^]^ Moitra et al. reported a Sb_2_S_3_‐based Huygen's metasurface with the capability of wavefront control at visible range (Figure 7i).^[^ ^160^ ^]^ The nanohole structure is adopted for the convenience of crystal nucleus growing and the consideration of Huygen's condition. For the aim of beam steering, periodical supercells consisting of eight nanoholes with diameters varying from 260 to 320 nm are constructed, as illustrated in SEM images in Figure 7i. There is a π/4 phase difference between the adjacent holes within the supercell at the working wavelength (≈705 nm). Consequently, an accumulated 2π phase difference is obtained for a supercell. Figure 7j displays the diffraction angle‐dependent normalized transmission spectra. For the amorphous state, beam deflection is observed at ≈705 nm, which corresponds to the +1 order diffraction. While for the crystalline state, the diffraction strength is extremely weak and is negligible compared to the 0 order mode, equivalent to no beam bending after crystallizing. Besides, the +1 order diffraction reemerges after the laser‐induced amorphization process, demonstrating the reconfigurable functionality in light manipulation.

In addition, chalcogenides have been demonstrated to achieve tunable hyperbolic metamaterials.^[^ ^161^, ^162^ ^]^ Behera et al. reported an optically reconfigurable hyperbolic metamaterial composed of alternately stacked Au and GST layers (Figure 7k).^[^ ^161^ ^]^ Due to its 2D planar configuration, the suggested hyperbolic metamaterial can also be considered as a hyperbolic metasurface.^[^ ^163^ ^]^ The lithography‐free multilayer structure facilitates large‐area and high throughput fabrication of the hyperbolic metamaterial. For the GST layers in the amorphous state, the resonant absorption peak of the hyperbolic metamaterial appears at 1180 nm (right panel in Figure 7l). After annealing at 200 °C for 30 min, the GST layers crystallize and the resonance peak redshifts to 1680 nm (middle panel in Figure 7l). Moreover, as depicted in Figure 7l (left), the application of the pulsed laser to induce melt‐quench causes a blueshift of the resonant wavelength to 1280 nm, illustrating the reamorphizing process of the hyperbolic metamaterial.

### 3.2 Reconfigurable On‐Chip Photonic Devices

#### 3.2.1 Electrically Reconfigurable On‐Chip Integrated Photonic Elements

**Figure 8:**
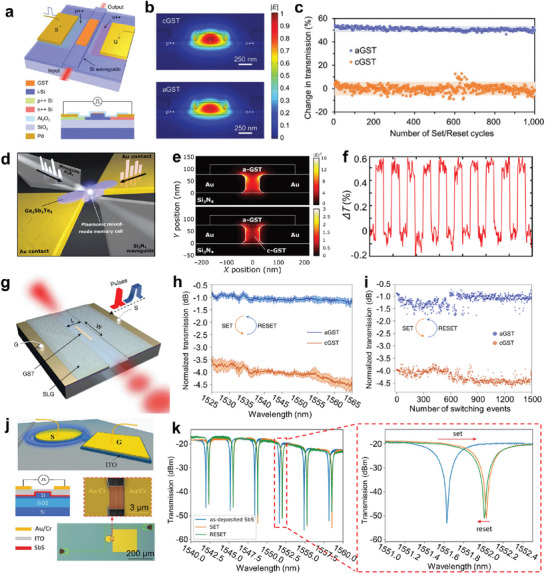
Electrically reconfigurable non‐volatile photonic devices. a) Schematic diagram of the GST‐on‐Si waveguide switch electrically modulated by Si PIN diode heater. The composed materials are labeled in different colors. b) Simulated electrical field intensity distribution profiles for fundamental quasi‐TE mode at 1550 nm when GST in crystalline (top) and amorphous (bottom) states of the waveguide switch. c) Cyclability plot of transmission change of the switch at 1550 nm during set and reset processes. The blue and orange shaded regions denote the two‐standard‐deviation intervals for amorphous and crystalline states. Reproduced with permission.^[^ ^100^ ^]^ Copyright 2020, Wiley‐VCH. d) Schematic illustration of the plasmonic nanogap enhanced phase change memory device. e) Simulated electrical field profile cross sections of the device when GST within the nanogap in amorphous (top) and crystalline (bottom) states. f) Time‐dependent transmission change of the device at 1570 nm during the cyclic switch between crystalline and amorphous states. Reproduced under the terms of the CC‐BY Creative Commons Attribution 4.0 International license.^[^ ^92^ ^]^ Copyright 2019, The authors, published by American Association for the Advancement of Science. g) Schematic diagram of the reconfigurable photonic switch based on GST. h) Reversible switching of the transmission spectra during GST phase change processes. The shaded areas denote the standard deviation during the set and reset cycles. The transmission spectra are normalized to the spectrum of a bare waveguide. i) Cyclability plot of transmission change of the photonic switch for 1500 switching. Reproduced with permission.^[^ ^172^ ^]^ Copyright 2022, Springer Nature. j) Schematic and optical microscope images of the Sb_2_S_3_‐based microring resonator with strong phase modulation. k) Measured transmission spectra of the microring switch in different states. Reproduced with permission.^[^ ^173^ ^]^ Copyright 2021, Wiley‐VCH.

Initially, GST was exploited as a data storage material for its reconfigurability and non‐volatility. Once switched, the resulting phase state can be maintained without an external energy supply.^[^ ^66^, ^67^ ^]^ With the development of integrated photonics, on‐chip PCMs photonic devices are required for prolonged or instantaneous information memory in compact optical storage, computing, and programming,^[^ ^164^, ^165^, ^166^, ^167^, ^168^, ^169^, ^170^, ^171^ ^]^ in which nanoscale optical waveguides and ring resonators are adopted as the constituted elements. Under the circumstances, the optical transmission of the waveguide is artificially controlled to represent different data levels. Electrically driving GST phase change to modulate the intensity of waveguide output light is a feasible scheme to achieve integrated non‐volatile photonic devices.^[^ ^106^, ^171^ ^]^ Zheng et al. demonstrated a non‐volatile electrically reconfigurable optical switch, which can be applied to non‐volatile photonic circuits.^[^ ^100^ ^]^ As shown in **Figure** **8**a, the switch adopts a GST‐on‐Si waveguide structure configuration and the Si PIN diode (p‐type, intrinsic, n‐type junction) heater acts as the external thermal source to stimulate phase transition by applying an electrical pulse. Single‐mode transverse (TE) electromagnetic wave transmission is supported by the Si waveguide. Figure 8b shows the simulated electrical field profile cross sections of the waveguide for quasi‐TE mode at 1550 nm, in which the electrical field intensity distribution of crystalline state is different from the amorphous one, indicating strong mode modification and subsequent propagation modulation of the switch unit. Transmission output spectra experimentally demonstrate the waveguide can be reversibly tuned. Specifically, multiple stable cycles of transmission state transformation by applying set/reset pulses over 1000 times imply an excellent endurance of the device is achieved (Figure 8c).

An electrical threshold switching scheme to attain phase change photonics memory was proposed by Farmakidis et al. (Figure 8d).^[^ ^92^ ^]^ The Au electrode tips form a plasmonic nanogap to enhance the inside field and the Au‐GST‐Au memory cell can couple to the Si_3_N_4_ waveguide to modulate the light transmission. By applying an electrical pulse, the GST phase inside the nanogap can be reversibly switched. Simulated electrical field intensity profiles show that the field enhancement is stronger for the amorphous state owing to the lower optical loss (Figure 8e). Although the light absorption is higher for c‐GST, the coupling is increased when GST is in the crystalline phase because of its larger refractive index, causing the relatively higher transmission from ≈1480 nm to 1600 nm. By alternately applying set and reset electrical pulses, time‐dependent optical transmission change of the Si_3_N_4_ waveguide at 1570 nm is obtained (Figure 8f), demonstrating the stable binary operations with reversible phase changes. Apart from that, the phase of GST in the nanogap can also be manipulated by laser pulses, thus a non‐volatile all‐photonics memory device is obtained, which will be detailly discussed in the later section.

Low energy consumption and high device endurance are of great importance for RMNODs for practical application. Fang et al. reported a non‐volatile electrically reconfigurable Si photonic switch with ultra‐low energy consumption based on the graphene heater (Figure 8g).^[^ ^172^ ^]^ The photonic switch consists of a Si ridge planarized waveguide and an upper GST strip, which are interspaced by the single‐layer graphene (SLG) electrical heater. Several Al_2_O_3_ layers are introduced as the protective layers. Through trigging GST phase change with electrical pulses, the optical mode in the GST‐clad waveguide is modified, resulting in modulation of the transmission amplitude. As illustrated in Figure 8h, the transmission spectra of the waveguide can be reversibly switched with bistable light signal output, covering the communication wavelength of 1550 nm. The transmission contrast is ≈3 dB. The amorphization energy density is calculated as 8.74 ± 1.42 aJ nm^−3^, showing extremely low programming energy consumption. A device endurance investigation is conducted by repeatedly switching the states up to 1500 times, which yields satisfactory results (Figure 8i). Besides, a micro‐ring resonator based on Sb_2_Se_3_ modulation to achieve phase shift is also presented.

Sb_2_S_3_‐based tunable optical devices have inherent superiority in the visible operating waveband. Employing the low‐loss chalcogenide Sb_2_S_3_, Fang et al. demonstrated a reconfigurable on‐chip microring switch with electrically tunable spectral shift.^[^ ^173^ ^]^ The designed switch adopts a coupled waveguide‐ring resonator structure configuration with a portion of the microring covered by the conformal Sb_2_S_3_ film (Figure 8j). An ITO electrical heater is patterned on the top of the Sb_2_S_3_ for active tuning. Figure 8k exhibits the wavelength‐dependent transmission of the microring switch, where several resonance dips with high‐quality factors are observed. After Sb_2_S_3_ crystallizing (SET operation), the TE mode inside Si microring is modified, which results in strong phase modulation. Consequently, a redshift of the resonance dip is observed. Then, a RESET operation is carried out by applying a 6 V, 200 ns electrical pulse, yielding a slight blueshift. The incomplete restoration of the transmission state is attributed to the nonuniform heating, which is expected to improve by optimizing the heater configuration.

#### 3.2.2 Optically Reconfigurable On‐Chip Integrated Photonic Devices

**Figure 9:**
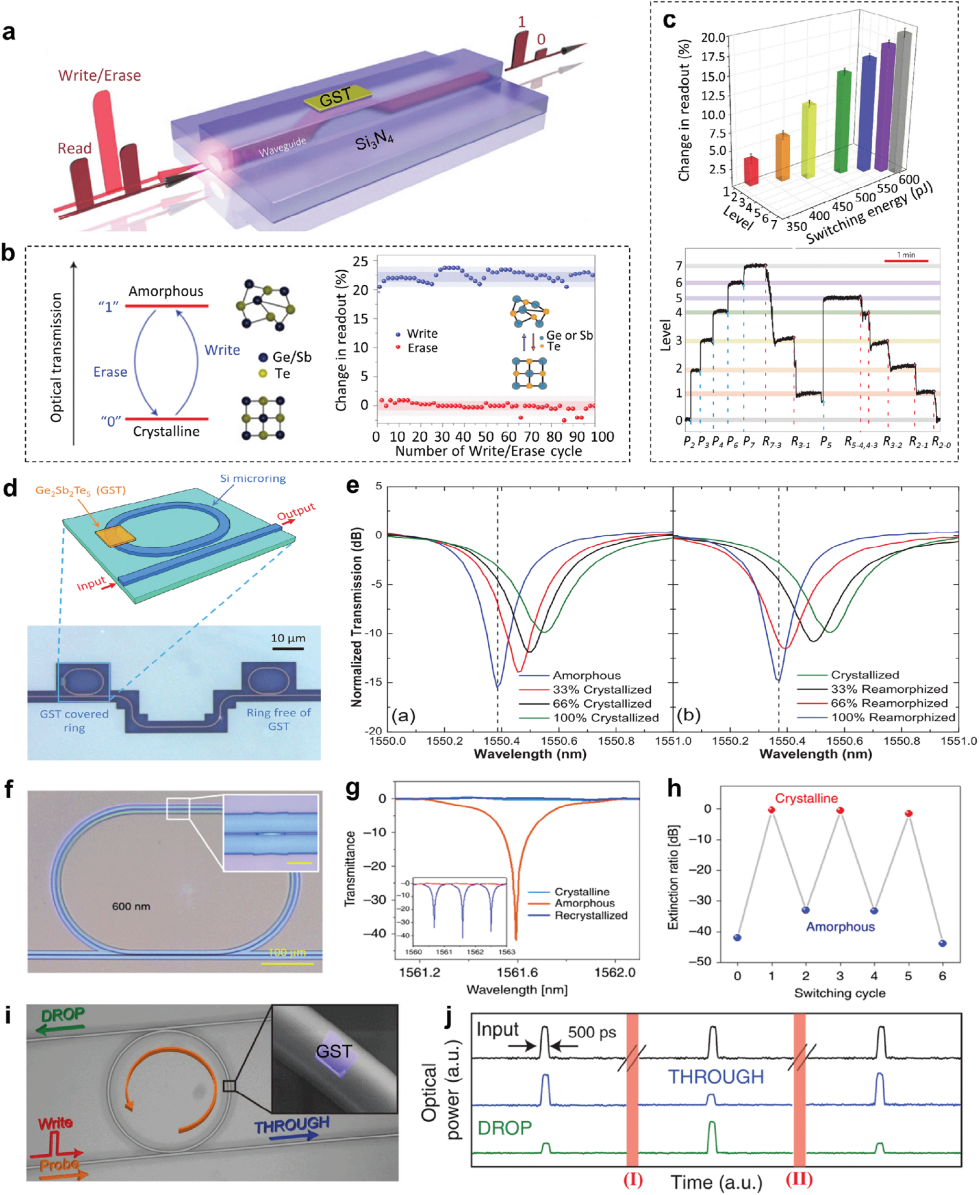
Non‐volatile reconfigurable all‐photonic devices. a) Schematic overview of the all‐photonic memory device with a nanoscale GST cell on the top of the waveguide. b) Binary memory operation between crystalline and amorphous state of GST (left). Multiple repetitions of the switching cycle for writing and erasing (right). Low optical transmission at crystalline state denotes “0”, and high optical transmission at amorphous state denotes “1”. c) Multi‐level memory operation accessed by intermediate crystallographic states of GST. Reproduced with permission.^[^ ^106^ ^]^ Copyright 2015, Springer Nature. d) Schematic illustration and optical microscope images of the optical microring switch based on GST. e) Normalized transmission spectra of the switch in different states of GST. Reproduced with permission.^[^ ^166^ ^]^ Copyright 2013, AIP Publishing. f) The optical microscope image of the optical switch consists of a resonator coupled to a waveguide. g) Normalized transmission spectra of the switch. The inset shows transmittance modulation in broadband. h) Resonance extinction ratio modulation of the device upon laser actuating. Reproduced under the terms of the CC‐BY Creative Commons Attribution 4.0 International license.^[^ ^108^ ^]^ Copyright 2019, The authors, published by Springer Nature. i) Optical microscope image of the reconfigurable all‐optical switch. j) Optical probing of both probe (black curve) and output (blue and green curves) pulse during the reversible modulation process. Reproduced with permission.^[^ ^178^ ^]^ Copyright 2016, Wiley‐VCH.

Optical information transmission is characterized by large bandwidth, high speed, and low crosstalk. It is desirable to calculate, transmit, and memorize information by all‐optical means.^[^ ^174^, ^175^, ^176^ ^]^ All‐photonic devices would reduce the latencies associated with electrical memory and potentially eliminate optoelectronic conversions, which is an extremely promising way to meet the high integration demand of photonic systems.^[^ ^174^, ^177^ ^]^ A scheme using Si_3_N_4_ waveguide covered by a GST cell to fulfill non‐volatile all‐photonic memory is shown in **Figure** **9**a.^[^ ^106^ ^]^ Both writing and erasing of the memory are performed by inducing GST phase transformation with the nanosecond laser. Sub‐nanosecond pulse or continuous light with low power is employed as the reading signal. The evanescent coupling between light propagating along the waveguide and GST is responsible for both writing into the GST memory cell and readout of the stored information. The c‐GST state has large ε_2_, leading to strong attenuation of the propagating light, which is defined as “0”. While a‐GST state allows more light to pass through for the relatively low ε_2_, producing a higher transmission and therefore defined as “1” (left panel in Figure 9b). After a repeated cycling of phase change, the transmittances of the reading signal basically maintain the original level (right panel in Figure 9b), demonstrating good reversibility. Apart from the binary data memory, multi‐level memory can be realized by tailoring the degree of crystallization of GST. The partial crystalline states are obtained by implementing a laser pulse with varying energy. There are seven distinct types of levels and the conversion among these levels is free of constraint (Figure 9c). As a result, this is particularly meaningful for high‐density data storage.

A reconfigurable optical microring switch with a modulation depth of 12 dB working at 1550 nm is suggested (Figure 9d).^[^ ^166^ ^]^ The GST film with an area of 3 × 1.5 µm^2^ and a thickness of 20 nm is deposited on the Si microring. The active control of GST state switching is achieved by leveraging an external pulsed laser with an output wavelength of 975 nm. For the GST cell in the amorphous state, a resonance appears at 1550.384 nm with a quality factor equal to 5656 (Figure 9e). As the crystalline degree of GST increases, the resonance dip gradually redshifts (1550.552 nm for 100% crystallized state), and the quality factor decreases accordingly (3641 for 100% crystallized state), showing dynamic modulation of both frequency and strength of the optical resonance mode. In addition, the transmission spectrum of the microring resonator can be reset by reamorphizing operation, demonstrating the reversible state switching.

A resonator scheme for the all‐photonic switch is experimentally demonstrated using GSST (Figure 9f), which possesses a high figure‐of‐merit (FOM), where FOM = Δn/Δk, Δn, and Δk denote the refractive index and extinction coefficient change after phase transition.^[^ ^108^ ^]^ The resonator, consisting of a SiN waveguide covered by GSST, accomplishes a non‐volatile photonic switch function. The normalized transmission spectra of the resonator in Figure 9g prove the optical contrast by controlling the resonant via phase transition. By dynamically switching the GST phase using laser pulses, the extinction ratio can be modulated reversibly, showing a large contrast ratio of 42 dB and a low insertion loss of less than 0.5 dB (Figure 9h).

All‐optical switches are crucial for establishing photonic circuits, as they offer a solution for avoiding photoelectric conversion. Stegmaier et al. reported a GST‐based non‐volatile all‐optical switch with tunable resonance coupling (Figure 9i).^[^ ^178^ ^]^ The all‐optical switch consists of a microring resonator and two evanescently coupled waveguides. Both the modulation (write) and probing light are introduced to the device via one of the waveguides. The probe light at resonance wavelength is divided into DROP and THROUGH ports, and the strength of the two components can be tuned through phase change of the GST cell. Figure 9j exhibits the input and output optical signal of the switch, where (I) and (II) denote amorphizing and crystallizing operations, respectively. After the amorphization of GST, the output light decreases by 4.7 dB for the THROUGH port and increases by 4.9 dB for the DROP port. Moreover, the transmission output can recover to the initial state by crystallizing the GST cell.

The reports mentioned above provide excellent experimental examples of reconfigurable on‐chip photonic devices. In addition, Pernice et al. theoretically proposed a photonic memory element that is based on a photonic microring resonator.^[^ ^179^ ^]^ To trigger the phase transition, a control port is introduced to couple with the GST section, through which a separate controlling light can be applied. Light propagating along the ring resonator couples evanescently with the GST. Accordingly, the GST state would have a significant effect on the propagating mode. There exists a range of sharp resonant dips for the amorphous state, where the resonance for the amorphous state features an optical quality factor up to 9400. When the GST turns to the crystalline state, the resonant becomes significantly weak due to enhanced optical loss.

### 3.3 Tunable Optical Film Devices

#### 3.3.1 Non‐Volatile Displays

**Figure 10:**
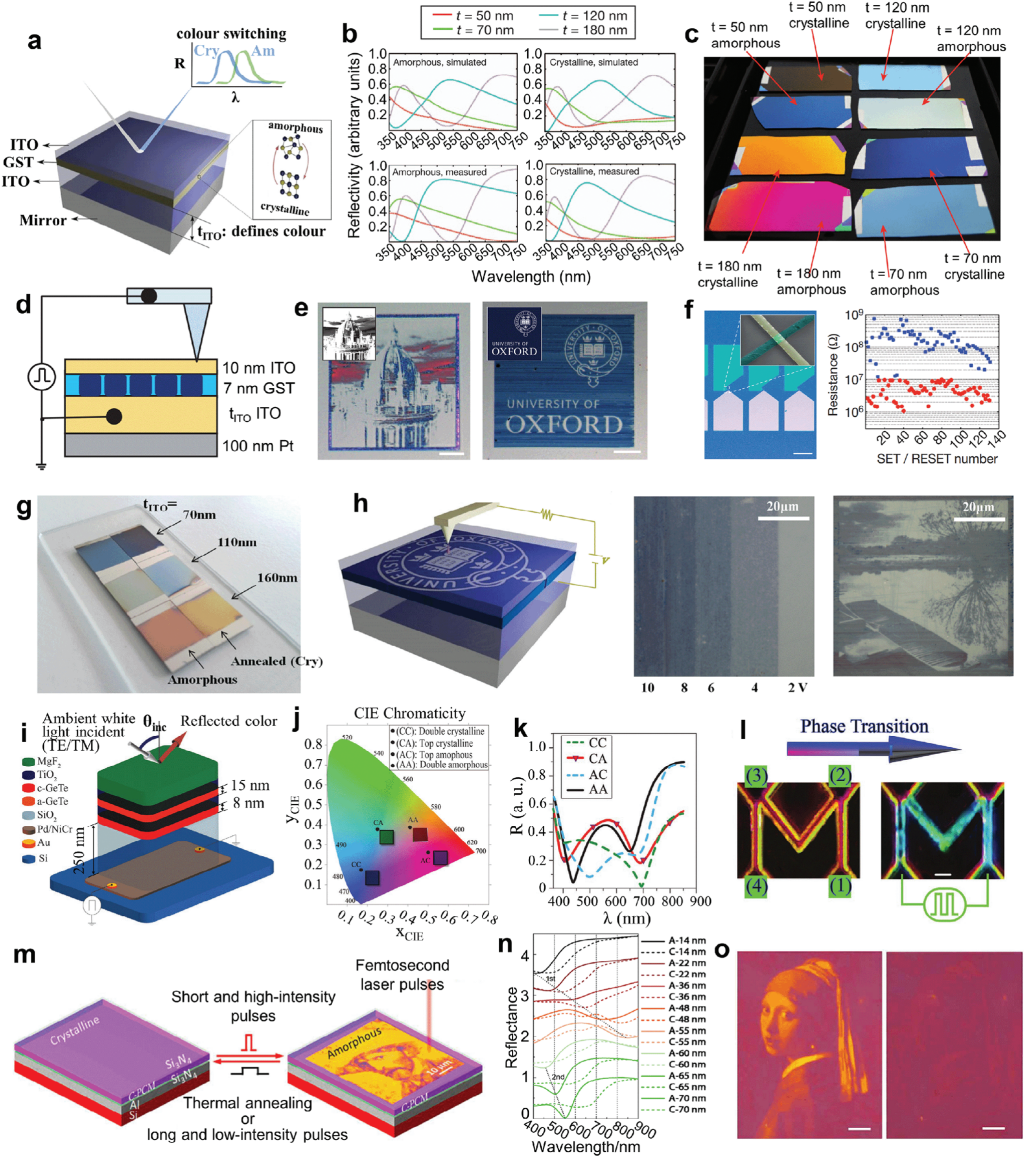
Tunable non‐volatile displays based on chalcogenide film. a) Schematic illustration of the multilayer structure consisting of stacked ITO/GST/ITO thin films. Reproduced with permission.^[^ ^191^ ^]^ Copyright 2016, Wiley‐VCH. b) Simulated (top) and measured (bottom) reflective spectra of the multilayer film structures with varying thicknesses of the bottom ITO layer. c) Photograph of the thin film samples comprising stacked 10 nm ITO/7 nm GST/*t* nm ITO/100 nm Pt mirror on the rigid substrates. The crystallization of GST is accomplished by heating samples to 220 °C for a few minutes. d) The electrical tuning scheme of the inducing GST film phase change by the conductive tip of an atomic force microscope (AFM). e) The optical microscope photos of the electrically constructed patterns on the multilayer samples. The insets on the left bottom indicate the original pictures of the writing patterns. f) Left: Optical image of vertical crossbar‐like ITO/GST/ITO devices with the overlapping area of 300 nm × 300 nm, and the inset shows the partially enlarged SEM image. The scale bar is 100 µm. Right: Electrical pulse‐driving cyclability plot of resistance of the fabricated device between set (crystalline) and reset (amorphous) states with low (red dots) and high resistance (blue dots), respectively. Reproduced with permission.^[^ ^188^ ^]^ Copyright 2014, Springer Nature. g) Photograph of the multilayer film devices for deep color modulation. The device comprising a stacked 10 nm ITO/7 nm AIST/*t_ITO_* nm ITO/100 nm Pt mirror is located on the SiO_2_ substrates, and *t_ITO_* denotes the thickness of the bottom ITO spacer film. The crystalline (Cry) sample is obtained by heating samples at 250 °C for 5 min. h) Left: Schematic illustration of the electrically induced phase change of local AIST film. Middle and right: The optical microscope photos of the electrical writing patterns on the 10 nm ITO/7 nm AIST/70 nm ITO samples. Reproduced with permission.^[^ ^191^ ^]^ Copyright 2016, Wiley‐VCH. i) Schematic rendering of the reconfigurable color reflector comprising GeTe. j) CIE chromaticity graph showing the four measured reflected colors corresponding to four different states of the device. Both GeTe layers crystalline, top layer crystalline and bottom layer amorphous, top layer amorphous and bottom layer crystalline, and both layers amorphous are described as CC, CA, AC, and AA, respectively. k) Reflection spectra of the reflector at different states for unpolarized light under 50° incident angle. l) Color transformation of the fabricated device with “M” sign shape when applying electrical pulse at port 1 and port 4 to drive GeTe phase transition. The scale bar corresponds to 10 µm. Reproduced with permission.^[^ ^104^ ^]^ Copyright 2019, Wiley‐VCH. m) Schematic diagram of the rewritable display based on Sb_2_S_3_. n) Measured reflection spectra of the device in amorphous (A) and crystalline (C) states. The thickness of the Sb_2_S_3_ film covers from 10 to 70 nm. o) Printing the desired pattern on the device using a femtosecond pulsed laser (left), and subsequently erasing the printed pattern through an annealing process (right). The scale bar is 20 µm. Reproduced under the terms of the CC‐BY Creative Commons Attribution 4.0 International license.^[^ ^105^ ^]^ Copyright 2020, The authors, published by American Association for the Advancement of Science.

Currently, the mainstream display technologies can be categorized as light‐emitting diode (LED), organic light‐emitting diode (OLED), and liquid crystal display (LCD).^[^ ^180^, ^181^, ^182^, ^183^, ^184^ ^]^ For a typical display with 400 pixels per inch (PPI), the feature size for a single pixel is ≈60 µm, which is far below the limitation allowed by the diffraction of light. Besides, the development of virtual reality (VR) displays and augmented reality (AR) glasses put forward higher standards on the resolution level.^[^ ^185^, ^186^ ^]^ Thus, a fundamentally novel device scheme is required to meet the rising demand for extremely small pixel size and ultra‐high pixel density in next‐generation displays.^[^ ^187^ ^]^ A non‐volatile optoelectronic display, taking advantage of phase transition‐induced reflective spectra variation, was proposed to fulfill nanometer‐scale color switching in multilayer films.^[^ ^188^ ^]^ As displayed in **Figure** **10**a, the multilayer structure consists of stacked 10 nm ITO/7 nm GST/*t* nm ITO films and the reflectivity mirror (Pt), where *t* denotes the thickness of the underneath ITO spacer film. The alteration of *t* would significantly affect the reflection spectra of the multilayer films and thus lead to different colors in visual (Figure 10b,c). After the crystallization of GST, the reflective spectra slightly change due to the refractive index discrepancy (Figure 10b), leading to apparent variations in colors (Figure 10c) for samples with the same *t*. To dynamically modulate the color, a nanometer‐sized electrical probe by AFM is introduced to electrically control the crystallization fraction within nanoscale areas by applying voltage, as illustrated in Figure 10d. The electrical drawing patterns are shown in Figure 10e, demonstrating the high optical contrast and the color‐rendering capability of the ultrathin‐film structure. Apart from rigid substrates, the flexible surface can also be used to support the image writing, suggesting a potential application in flexible wearable devices. Figure 10f (left) shows the crossbar‐like ITO/GST/ITO devices with overlapping crossbars of 300 nm × 300 nm. The GST at the overlapping area is cyclically transformed to the crystalline state by applying direct current and to the amorphous state by applying an electrical pulse with high amplitude, (Figure 10f (right)) demonstrating state switching of a nanoscale pixel is reversible and repeatable.

Notably, Sb_2_Te_3_/GeTe,^[^ ^189^ ^]^ Sc‐doped Sb_2_Te_3_,^[^ ^190^ ^]^ AIST,^[^ ^191^ ^]^ Sb_2_S_3_,^[^ ^105^ ^]^ and GeTe^[^ ^192^ ^]^ have also been demonstrated to achieve tunable color displays. For the material similarity of AIST and GST, an AIST‐based multilayer framework for deep color modulation is naturally proposed after the demonstration of GST colored display.^[^ ^191^ ^]^ Figure 10g shows the multilayer film structures comprising stacked 10 nm ITO/7 nm AIST/*t*_ITO_ nm ITO films and 100 nm Pt mirror. After the crystallization of AIST induced by annealing, the colors of the samples with the same *t* obviously change due to blueshifts of reflectivity curves. To electrically control the phase change in the desired nanoscale area, a conductive AFM is introduced to apply voltage bias between the ITO layers to generate Joule heat (left panel in Figure 10h). Degree of crystallization of the ASIT film increases with the increasing amplitude of the applied voltage, showing a deep grayscale modulation (middle panel in Figure 10h). Thus, by mapping the gray level scale to the voltage amplitude, a grayscale image is obtained through AFM writing (right panel in Figure 10h). Due to the same design philosophy and structure configuration, the erasing (amorphizing) and rewriting (recrystallizing) operation of nanoscale AIST pixels are accessible by implying the on‐chip cross‐bar devices presented in Figure 10f.

GeTe, as another PCM belonging to chalcogenides, has been demonstrated to achieve the reconfigurable color reflector by selective phase change in a multilayer structure device (Figure 10i).^[^ ^104^ ^]^ The multilayer device contains two GeTe layers with different thicknesses. The bottom Pd/NiCr film serves as the cavity mirror as well as the metal heater. The top MgF_2_ and TiO_2_ layers together act as the antireflection layer to increase the color contrast. Similarly, the separation of GeTe from the bottom metal layer by SiO_2_ can increase the color contrast as well. When the film thickness is less than ≈20 nm, the crystallization temperature of the GeTe layer increases as the film thickness decreases, which is an essential condition to separately control the phase states of the two GeTe layers. By applying an electrical pulse with different parameters, the device can be tuned from the original state (AA) to other states (CC, CA, and AC), which have distinct reflected colors with each other (as shown in Figure 10j). Moreover, the device can be reset to AA state by applying a voltage pulse of 20 V, revealing the reversible modulation. Reflection spectra of the reflector at different states with 50° angle of incidence are displayed in Figure 10k, showing the reflection response variations induced by phase transitions. Figure 10l illustrates the “M” sign shape device with the original red‐pink color changing to blue color after GeTe phase transition induced by an electrical pulse, corresponding to transitioning AA state to CC state.

Both the above electrical threshold method (conductive AFM) and the external electrical heater scheme exemplify the effectiveness of non‐volatile chalcogenide displays with ultra‐small pixels. Meanwhile, the laser direct writing technique offers an alternative approach for achieving localized phase transition and thus printing the desired patterns on the chalcogenide films. Liu et al. demonstrated a femtosecond laser‐printing rewritable color display with an ultra‐high pixel density.^[^ ^105^ ^]^ As depicted in Figure 10m, the device employs a multilayer configuration of Si_3_N_4_/Sb_2_S_3_/Si_3_N_4_/Al, with the two Si_3_N_4_ layers serving as the protective layer and the diffusion barrier, respectively. Sb_2_S_3_ is selected due to its significant contrast in refractive index between amorphous and crystalline states, as well as its relatively low extinction coefficient in the visible band. After subjecting the crystalline device to femtosecond laser pulses with adjustable output power, a spatially varying distribution of the degree of amorphization in Sb_2_S_3_ is obtained, which yields the color image. In addition to the phase state of Sb_2_S_3_, the reflection color is also influenced by the thickness of the Sb_2_S_3_ film (Figure 10n). To demonstrate the laser‐printing scheme, a color image is written on the device with 15 nm thick Sb2S3 film (left panel in Figure 10o). Moreover, the printed image can be conveniently erased through annealing (right panel in Figure 10o), facilitating the rewriting operation.

#### 3.3.2 Tunable Fano‐Resonant Optical Coatings

**Figure 11:**
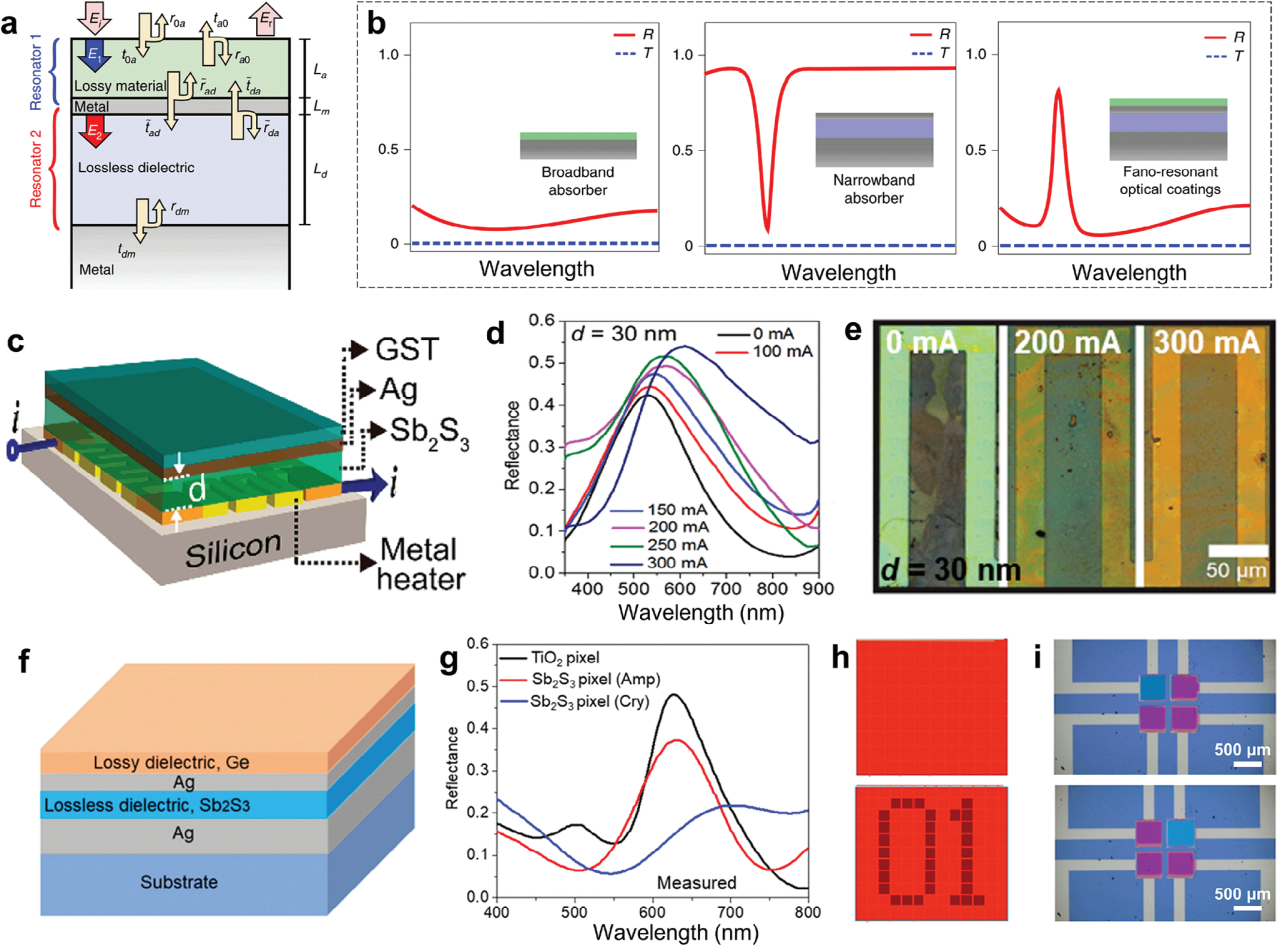
Tunable FROCs based on chalcogenide films. a) Schematic illustration of the structure of the FROCs. b) The reflection and transmission spectra of the broadband, narrowband absorber, and FROCs. Reproduced with permission.^[^ ^197^ ^]^ Copyright 2021, Springer Nature. c) Schematic diagram of the tunable thin film optical coatings based on GST and Sb_2_S_3_. d) Measured reflection spectra of the tunable optical coating under various DC currents. e) Optical microscope image of color change in the tunable FROC upon application of DC currents. Reproduced with permission.^[^ ^198^ ^]^ Copyright 2021, American Chemical Society. f) Schematic of the tunable Fano‐resonant nano‐optical coatings. g) Measured reflection spectra of the tunable FROC and the static FROC with TiO_2_ as the lossless dielectric medium. h) Schematic diagram of the steganographic reflector with multiple pixels before (top) and after (bottom) applying external stimulation. i) Image of the 2 × 2 pixel array of the electrically tunable FROC placed on the electrical microheater. Reproduced with permission.^[^ ^199^ ^]^ Copyright 2023, American Chemical Society.

Fano resonance occurs when a discrete localized state becomes coupled to a continuum of states.^[^ ^193^ ^]^ Over the past few years, the Fano resonance has been observed in metasurfaces containing plasmonic or dielectric nanostructures.^[^ ^194^, ^195^, ^196^ ^]^ Recently, the ultrathin film optical coatings with Fano resonance features were proposed by ElKabbash et al. (**Figure** **11**a).^[^ ^197^ ^]^ The Fano‐resonant optical coating (FROC) consists of stacked lossy material/metal/lossless dielectric/metal film layers, where the first two layers and last three layers constitute the resonators 1 and 2, respectively. The resonator 1 exhibits a broadband absorption spectrum (i.e., the continuum) while the resonator 2 presents a narrowband absorption peak (i.e., the discrete state), as depicted in the left and middle panels in Figure 11b. The coupling between the two resonators results in destructive interference between the constant and steep varying phase of the overlapping broadband and narrowband resonances, and thus lead to a narrow reflection peak that exhibits the asymmetric Fano line shape, as shown in Figure 11b (right).

As previously illustrated, in the VIS‐NIR region, Sb_2_S_3_ exhibits lower optical loss compared to GST due to its wider bandgap. Therefore, it is reasonable to employ Sb_2_S_3_ as the lossless dielectric and GST as the lossy material to achieve chalcogenide‐based tunable FROCs. As shown in Figure 11c, a Fano‐resonant thin film optical coating with electrical tunability was reported by Sreekanth et al.^[^ ^198^ ^]^ The tunable FROC adopts the stacked 20 nm GST/20 nm Ag/*d* nm Sb_2_S_3_/metal heater. The line width and resonance wavelength of Fano resonance can be adjusted by changing the thickness of the Sb_2_S_3_ layer. For the configuration of *d* = 30 nm, the asymmetric Fano‐resonant peak of the FROC in the amorphous state (0 mA) is located at ≈530 nm (Figure 11d). After applying DC current to the bottom metal heater, the resonant wavelength gradually redshifts with the increase of the current, which can be attributed to the crystallization of the chalcogenide films. Besides, the color of the FROC changes from green to yellow correspondingly (Figure 11e).

Another tunable FROC with a similar structure configuration has been demonstrated by Prabhathan et al., as shown in Figure 11f.^[^ ^199^ ^]^ The nano‐optical coating adopts the standard FROC structure, where the upper resonator (20 nm Ge/20 nm Ag) with a broad absorption band is stacked on the lower resonator (20 nm Ag/50 nm Sb_2_S_3_/100 nm Ag) with a narrow absorption band for coupling. The measured reflection spectra of the tunable FROC are depicted in Figure 11g. The reflection spectra of the non‐tunable FROC, where Sb_2_S_3_ is substituted with a TiO_2_ cavity, are also provided for comparison. Adjusting the TiO_2_ cavity thickness would aligns the equivalent optical path lengths, yielding identical Fano‐resonant wavelengths for both FROCs. (Figure 11g). After applying external stimulation, the resonance peak of the tunable FROC pixels redshifts while the counterpart with TiO_2_ cavity remains unchanged, thus lead to the realization of the steganographic reflector (Figure 11h). Moreover, the individual tuning of FROC pixels by DC current has also been demonstrated (Figure 11i).

The two chalcogenide‐based optical coating schemes presented above demonstrate the effective use of phase change materials in achieving tunable FROCs. Considering the device compatibility between the DC current heater and pulsed electrical heater, it is feasible to realize reversible modulation by applying high energy short electrical pulses.

## 4 Vanadium Dioxide (VO_2_)

### 4.1 VO_2_‐Based Photodetectors

Photodetectors are devices that finely respond to external irradiation by converting a light signal into an electrical signal, showing extremely valuable applications in sensing, imaging, environment monitoring, security checking, and smart response devices.^[^ ^200^, ^201^, ^202^ ^]^ According to the triggering mechanism of photocurrent, photodetectors can be divided into two types. The one type is associated with the effects of photon‐generated free carriers, including photovoltaic, photoconductive, and photogating effects. The other is attributed to the thermal effect, including photothermoelectric and bolometric effects.^[^ ^203^ ^]^ The bandgap values of photoresponsive materials significantly determine the suitable working waveband of their based detectors. Responsivity and detectivity are two essential parameters to characterize the performance of the photodetector. Responsivity (*R_λ_*) denotes the photocurrent generated per unit power of light on the effective area of the detector, *R_λ_ = I_ph_/(PS)*, where *I_ph_* is the difference value between photocurrent and dark current, *P* is the light power density, and *S* is the effective illuminated area. Specific detectivity (*D^＊^*) is used to evaluate the detector sensitivity, *D^＊^ = R_λ_S^1/2^/(2eI_d_)^1/2^*, where *R_λ_* is the responsivity, *S* is the effective illuminated area, *e* is the electronic charge, and *I_d_* is the dark current. Photodetectors based on silicon,^[^ ^204^ ^]^ InGaAs,^[^ ^205^ ^]^ HgCdTe,^[^ ^206^ ^]^ InSb,^[^ ^207^ ^]^ SiAs,^[^ ^208^ ^]^ InAs/InGaSb,^[^ ^209^ ^]^ and 2D materials (e.g., graphene,^[^ ^210^ ^]^ black phosphorous,^[^ ^211^ ^]^ transition metal dichalcogenides,^[^ ^212^, ^213^ ^]^ tellurium,^[^ ^214^ ^]^ boron nitride^[^ ^215^ ^]^) have been widely investigated, and some of them have become a prominent part in the commercial market. Transition metal oxides provide another choice to accomplish the goal of light detection, in which VO_2_ is a typically representative and worth intensively studying. Benefitting from photoconductive, bolometric effect, or photoinduced phase transition effect, VO_2_‐based photodetectors could be switched between high and low resistance states and thus detect light in high efficiency ways.^[^ ^156^, ^216^, ^217^, ^218^, ^219^ ^]^

**Figure 12:**
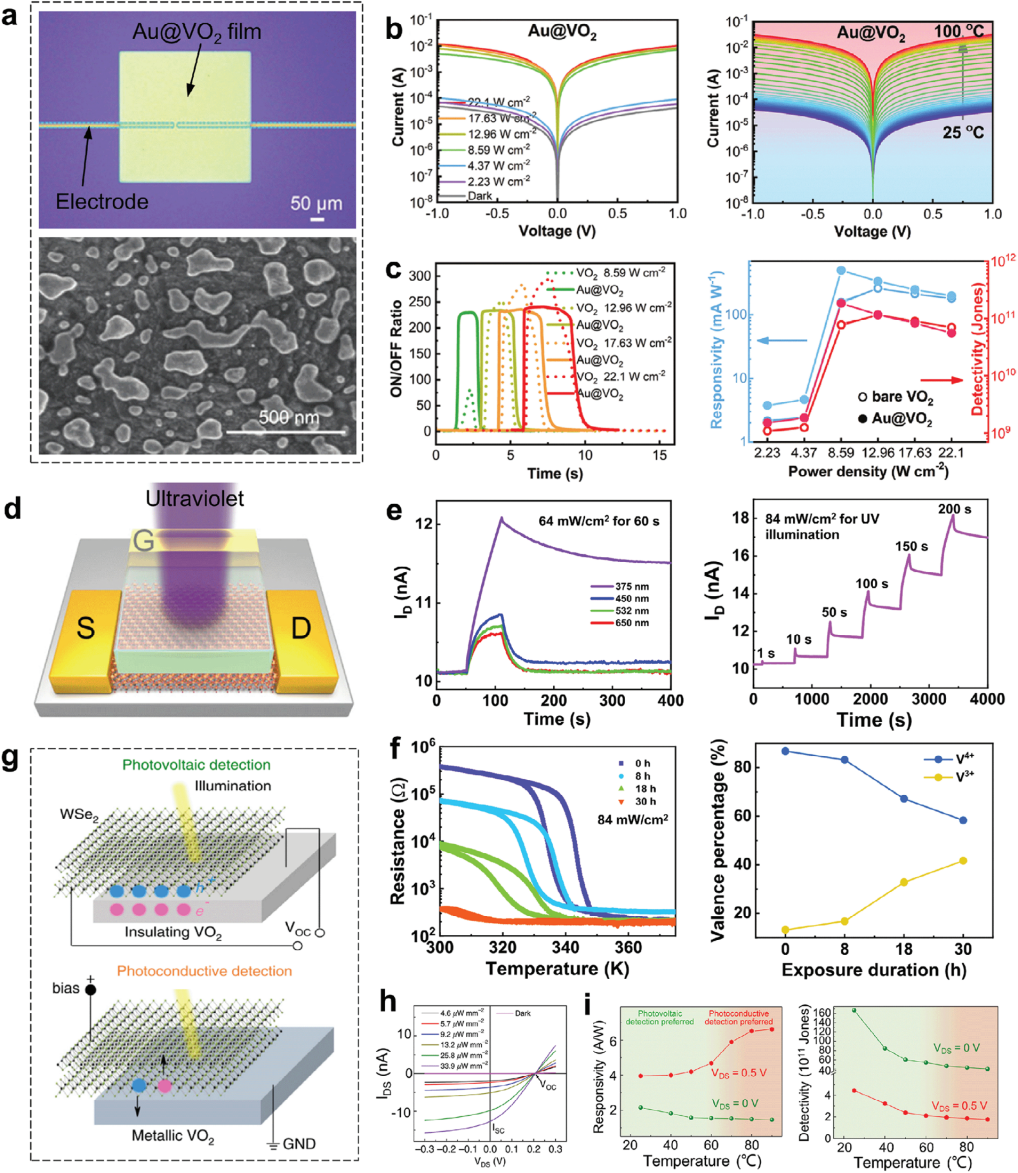
VO_2_ thin film photodetectors working at various wavebands. a) Optical microscope images of the Au@VO_2_ IR photodetector (top) and SEM image of Au@VO_2_ film (bottom). b) *I‐V* curves of the Au@VO_2_ IR photodetector under different irradiation conditions at room temperature (left). Temperature‐dependent *I‐V* curves of the Au@VO_2_ IR photodetector (right). c) Photocurrent response of the photodetectors at a bias of 1 V under different power densities (left). Power density‐dependent responsivity and detectivity of the photodetectors (right). Reproduced with permission.^[^ ^216^ ^]^ Copyright 2021, Wiley‐VCH. d) Schematic of VO_2_ detector for UV sensing. e) Left: Responses of the drain current to illumination with wavelengths at 375 nm (ultraviolet), 450 nm (blue), 532 nm (green), and 650 nm (red). Right: Responses of the drain current to ultraviolet irradiation with duration time for 1 s, 10 s, 50 s, 100 s, 150 s, and 200 s. f) Temperature dependence of VO_2_ film resistance curves at various illuminating durations of ultraviolet with the intensity of 84 mW/cm^2^ (left). The integrated area percentage of vanadium valence peaks at different exposure durations of ultraviolet light (right). Reproduced under the terms of the CC‐BY Creative Commons Attribution 4.0 International license.^[^ ^156^ ^]^ Copyright 2022, The authors, published by Springer Nature. g) Schematic of the dual‐mode WSe_2_/VO_2_ heterojunction photodetector. h) *I–V* characteristics of the WSe_2_/VO_2_ photodetector under illumination with different power densities at room temperature. i) Temperature‐dependent responsivity (left) and detectivity (right) of the photodetector under 0 and 0.5 V external bias. Reproduced with permission.^[^ ^219^ ^]^ Copyright 2019, AIP Publishing.

Taking full advantage of the MIT of VO_2_ induced by light radiation is an advisable strategy to detect photons. A phase transition‐triggered Au@VO_2_ IR photodetector has been suggested (**Figure** **12**a).^[^ ^216^ ^]^ The photodetector is designed with an Au@VO_2_ film structure configuration, where irregular Au nanoislands with sizes ranging from 5 to 200 nm are located on the VO_2_ film. The IR photodetection response is improved by LSPR‐assisted hot electron injection. As shown in the left panel in Figure 12b, the photocurrent increases apparently with the increase of IR illumination power intensity. The temperature‐dependent I‐V curves show that the heating has the same effect as the IR light (right panel in Figure 12b), indicating light absorption‐induced phase change of VO_2_ film. To demonstrate the superiority of the Au@VO_2_ structure, a bare VO_2_ film photodetector is fabricated as a comparison. In fact, the Au@VO_2_ photodetector has a relatively high response speed and a much steady saturated ON/OFF ratio, as given in Figure 12c (left). In addition, the responsivity and detectivity of the Au@VO_2_ photodetector are displayed in Figure 12c (right), indicating excellent IR detection performance.

A VO_2_ detector for UV sensing was reported based on the anomalous non‐volatile phase transition of VO_2_.^[^ ^156^ ^]^ The VO_2_‐based UV detector adopts an optoelectronic transistor structure, where VO_2_ film acts as the channel between source (S) and drain (D) electrodes and VO_2_ film serves as gating (G) (Figure 12d). The incident UV plays the role of gating modulation, stimulating M‐VO_2_ changes to R‐VO_2_. Under the exposure of light with various wavelengths (375 nm, 450 nm, 532 nm, and 650 nm) at an intensity of 64 mW/cm^2^, the drain currents (*I_D_*) increase apparently, as exhibited in Figure 12e (left). When withdrawing the illumination, only the transistor exposed to UV light shows obvious non‐volatility, while those under visible light are volatile. Figure 12e (right) shows the stepwise increase of I_D_ under UV irradiation with different duration time (1 s, 10 s, 50 s, 100 s, 150 s, and 200 s), indicating the value of non‐volatile I_D_ increases along with the increase of exposure time. Moreover, the temperature‐dependent resistance of VO_2_ film under UV illumination with various exposure durations further confirms the abnormal non‐volatile transition (left panel in Figure 12f). The volatile photoresponse to visible light is attributed to the photoconductive effect, namely photoinduced carriers cause the increase of free carrier concentration and it would decrease after the termination of the irradiation. While non‐volatile behavior is caused by the UV light‐induced phase transition. The proportions of V^3+^ and V^4+^ states show opposite changes with the increase of exposure duration (right panel in Figure 12f), indicating a proportion of ≈28.5% vanadium ions’ chemical environment change and from which the concentration of oxygen vacancies was estimated as 4.85 × 10^21^ cm^−3^. Thus, the UV light‐induced oxygen vacancies cause the metallic phase transition and suppress the volatility. For the reset process, electrolyte gating is adopted to insert oxygen ions into the VO_2_ crystal lattice, which decreases the oxygen vacancies and gradually transitions R‐VO_2_ back to M‐VO_2_.

Another VO_2_‐based photodetector with high performance is demonstrated by Luo et al.^[^ ^219^ ^]^ As illustrated in Figure 12g, the VO_2_ film is covered by WSe_2_ flakes, which form a heterojunction. Photovoltaic and photoconductive effects dominate the generation of photocurrent in the devices for VO_2_ film in insulating (room temperature) and metallic (heating) states, respectively. Figure 12h presents the *I–V* curves of the WSe_2_/VO_2_ photodetector under illumination (LED source) with different power densities at room temperature, revealing a dramatic photovoltaic behavior. The photovoltaic effect yields a large built‐in electric field, which effectively separates the photoexcited electron‐hole pairs and forms a type‐II heterojunction. The open‐circuit voltage (*V_OC_*) and short‐circuit current (*I_SC_*) are measured as 208 mV and 12.7 nA, respectively. Moreover, at the illumination of 4.6 µW mm^−2^, the *R_λ_* = 2.4 A/W and *D^＊^* = 1.87 × 10^13^ Jones are obtained, demonstrating the excellent photo‐response performance. After heating VO_2_ above the phase transition temperature, the barrier height at WSe_2_/VO_2_ interface declines to ≈0.1 eV, thus, the type‐II heterojunction transforms to Schottky junction. The lowered barrier height and the improved electrical conductance of VO_2_ dramatically enhance the injection efficiency of photoexcited carriers at the heterojunction interface. This results in a much larger photocurrent at a relatively low bias. Figure 12i shows the temperature‐dependent responsivity and detectivity of the WSe_2_/VO_2_ photodetector, exhibiting a large *R_λ_* of 2.4 A W^−1^ and an acceptable *D^＊^* of 1.77 × 10^11^ Jones under 0.5 V external bias at 90 °C.

The above three schemes based on the phase transition of VO_2_ film provide innovative ideas to achieve the goal of photodetection. Some other VO_2_‐based photodetectors deserve to be introduced as well. In 2004, Li et al. first demonstrated the photoconductive VO_2_ detectors for NIR light. The NIR detector is composed of the inner single‐domain M‐VO_2_ core and the outer V_2_O_5_ shell, constituting core/shell nanobeam heterostructures.^[^ ^218^ ^]^ The nanobeam structure produces a high surface‐to‐volume ratio, leading to ultrahigh detection sensitivity. In addition, benefiting from the heterostructure of VO_2_/V_2_O_5_, the photon‐generated electrons in VO_2_ spontaneously transfer from VO_2_ to V_2_O_5_, prolonging the lifetime of excitons and building electron/hole conductive channels, and thus enhancing the photoconductivity as well as improving NIR detection ability. The maximum value of responsivity (*R_λ_*) and specific detectivity (*D^＊^*) is 2873.7 A W^−1^ and 9.23 × 10^12^ Jones at 0.2 mW cm^−2^ irradiation, respectively, proving the excellent performance compared with reported photodetectors based on other materials. Broadband photodetectors have the ability to detect incident light across a wide range of spectra. An ultra‐broadband Wadsley B phase VO_2_ (VO_2_ (B)) photodetector covering from visible to terahertz region (405 nm to 0.88 mm) is proposed by Zhang et al.^[^ ^217^ ^]^ VO_2_ (B) possesses a zero‐band gap, which makes it suitable for broadband photodetection. Besides, the large temperature coefficient of resistance, −4.77% K^−1^ at 40 °C, makes VO_2_ (B) an eligible material to achieve hypersensitive radiation detection based on the bolometric effect. The *D^＊^* of the device from visible to NIR range is greater than 1.79 × 10^12^ Jones under 1550 nm laser both in air and vacuum conditions.

### 4.2 All‐Optical Switches

**Figure 13:**
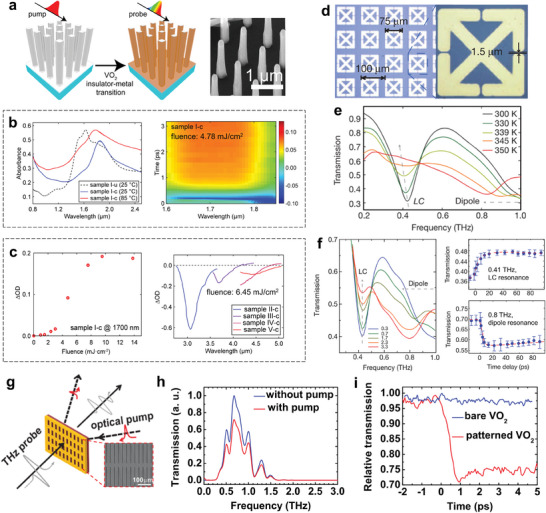
VO_2_‐based metasurfaces for all‐optical switching at infrared and terahertz regions. a) Schematic illustration of the VO_2_ conformal nanorod array for broadband all‐optical switching and corresponding SEM image of the fabricated sample. b) Measured absorption spectra of ΔD (change of optical density) for sample I‐u at 25 °C and sample I‐c at 25 °C and 85 °C (left). ITO nanorod arrays have a pitch size of 1 µm and nanorod height of 2 µm is denoted as sample I. Samples coated with VO_2_ and without coating are denoted as sample I‐c and sample I‐u, respectively. NIR transient spectral map of sample I‐c under a 1580 nm pump pulses with a fluence of 4.78 mJ cm^−2^ (right). c) Fluence dependence of ΔD for sample I‐c at 1700 nm averaged over a delay time from 2 to 3 ns (left). Transient spectra averaged over a delay time from 2 to 3 ns for samples with various structure parameters (right). The pump fluence is 6.45 mJ cm^−2^. Reproduced with permission.^[^ ^61^ ^]^ Copyright 2017, American Chemical Society. d) Optical image of the terahertz all‐optical switch with Au SRR on the VO_2_ film. The SRR gap is 1.5 µm. e) Transmission spectra of the metasurface under various temperatures with LC and dipole resonances. f) Transmission spectra of the metasurface under terahertz pump laser at the in‐gap field strength of 0.3 MV/cm, 0.7 MV cm^−1^, 1.7 MV cm^−1^, 2.3 MV cm^−1^, and 3.3 MV cm^−1^ (left). Transient terahertz probe measurements at the frequency of 0.41 THz and 0.8 THz (right). Reproduced with permission.^[^ ^222^ ^]^ Copyright 2012, Springer Nature. g) Schematic of the VO_2_ all‐optical switch at the terahertz region and its SEM image. h) Transmission spectra of the metasurface with and without pump pulse. i) Transient time‐dependent relative transmission of the sample with bare and patterned VO_2_ film with pumping at 0 ps. Reproduced with permission.^[^ ^223^ ^]^ Copyright 2011, AIP Publishing.

Hybridizing PCMs with plasmonic metastructures would trigger extraordinary optical modulation phenomena.^[^ ^220^, ^221^ ^]^ Specifically, the resonators in the hybrid structure can yield sub‐wavelength field enhancement to induce the phase transition of PCMs. In the meantime, the plasmonic resonances are actively modulated because of the abrupt change of the dielectric environment along with phase change. This scheme provides a competing platform to achieve the function of the all‐optical switch. The ultrafast phase change response (≈5 ps) and relatively low insulator‐metal transition (IMT) temperature (i.e., low energy consumption) make VO_2_ a unique material in the ultrafast switching process. Guo et al. demonstrated a conformal coating of an ultrathin VO_2_ film on the ITO nanorod array for broadband all‐optical switching (**Figure** **13**a).^[^ ^61^ ^]^ The phase transition of VO_2_ film is attributed to the excitation of NIR pump laser pulses. As a contrast, the static absorption spectra of samples under different temperatures are given in Figure 13b (left). The VO_2_ coating film causes a redshift of the LSPR in the ITO nanorod array. The IMT of VO_2_ leads to a blueshift for the decrease of the real part of the refractive index of VO_2_ film and the broadening of the LSPR for the damping of the resonance is caused by the oscillations of free electrons in R‐VO_2_. The transient spectral map (right diagram of Figure 13b) in the NIR region reveals the ultrafast response of VO_2_ phase change and the detailed changing process of optical density. Figure 13c (left) shows the fluence‐dependent ΔD at 1700 nm with pump pulses averaged over a delay time from 2 to 3 ns, demonstrating that the transient optical switching is ascribed to the phase transition of VO_2_. Besides, by adjusting the structure parameters of the nanorods, the switching response can be achieved in the MIR region (right panel in Figure 13c).

Apart from NIR laser pulses, terahertz pulses can also serve as the pump and have been proven to realize insulator to metal transition of VO_2_ (Figure 13d).^[^ ^222^ ^]^ The Au SRRs are placed on the VO_2_ films, enhancing the terahertz electric field to reduce the Coulomb‐induced potential barrier for carrier transport and thus induce electronic phase change of VO_2_. The transmission spectra of the device under various temperatures are displayed in Figure 13e. With the temperature increasing, the transmission at LC resonance increases and the resonance peak shows a redshift, which is attributed to the metallization‐induced increased conductivity of VO_2_ in SRRs gap and increased permittivity of VO_2_ film, respectively. The dipolar resonance exhibits a redshift and it's also the result of the conductivity increasing of the in‐gap VO_2_. As shown in Figure 13f (left), with the strengthening of the incident terahertz pump laser, the transmission spectra show consistent behaviors with the results in Figure 13e, indicating the terahertz field‐induced phase transition to R‐VO_2_. The transient terahertz probe measurements at LC and dipole resonance reflect the ultrafast dynamic response of the all‐optical switching process (right diagrams of Figure 13f).

The hybrid metasurfaces consisting of VO_2_ and plasmonic metastructures open up a creative way to all‐optical switching. In addition, the all‐dielectric patterned VO_2_ film has been demonstrated as a feasible approach to realize the all‐optical switch as well (Figure 13g).^[^ ^223^ ^]^ The VO_2_ film on the sapphire substrate is patterned with periodic slot arrays. The transmission spectra of the patterned VO_2_ metasurface with and without terahertz pump pulses are shown in Figure 13h, where a reduction of transmission excited by terahertz is observed. To estimate the optical response, the time‐dependent relative transmission spectra of the sample with bare and patterned VO_2_ film are measured (Figure 13i). The former shows no change while the latter decreases after excitation of terahertz pump pulses within 1 ps, exhibiting ultrafast modulation of terahertz transmission.

### 4.3 Tunable Metasurface Absorbers

**Figure 14:**
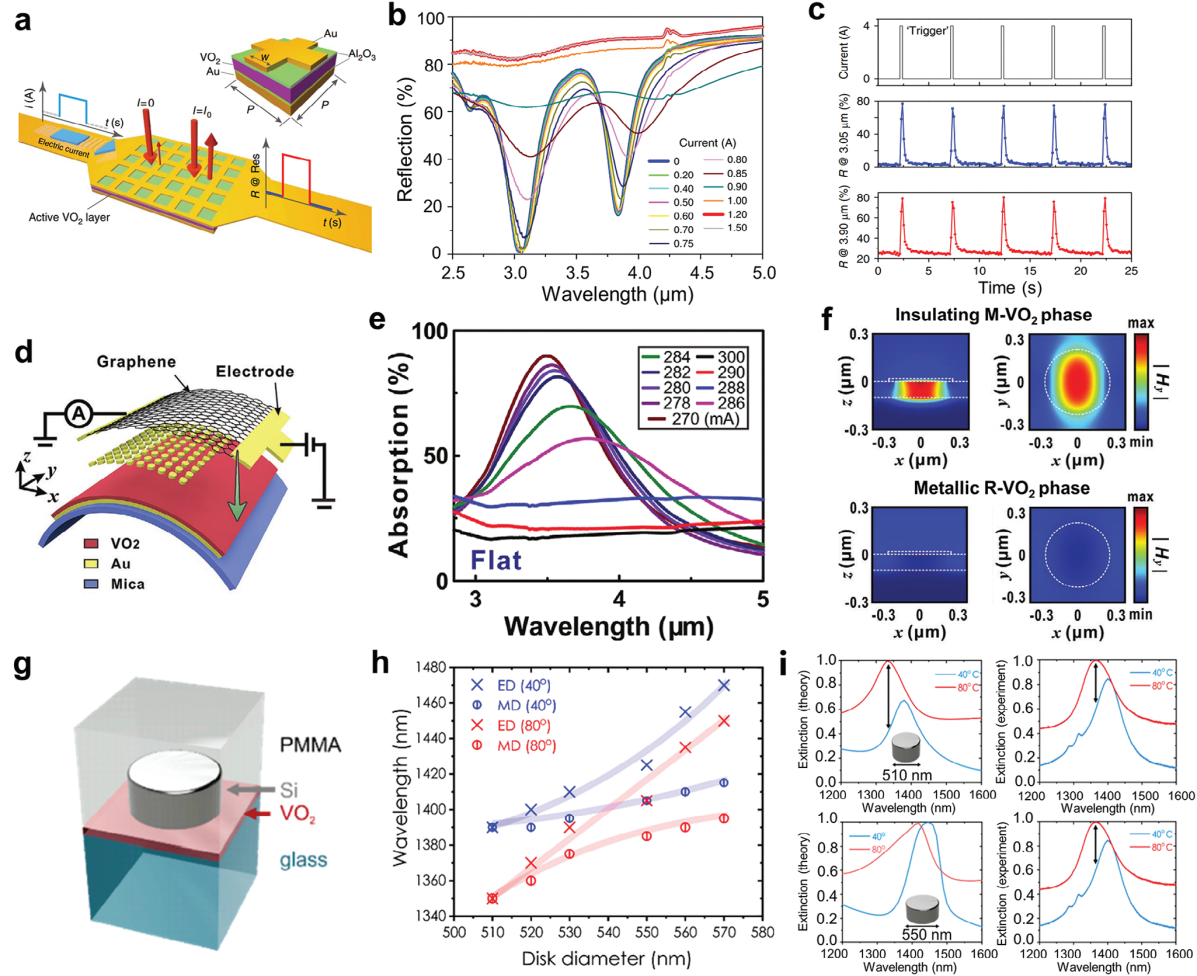
Tunable metasurface absorbers based on VO_2_. a) Diagram of the hybrid metasurface for electrically tunable absorption at the MIR region. b) The reflection spectra of the metasurface modulated by electrical current with various intensities from 0 to 1.5 A. c) Electrical switching of the reflection at resonant absorptions of 3.05 µm and 3.9 µm. Reproduced under the terms of the CC‐BY Creative Commons Attribution 4.0 International license.^[^ ^93^ ^]^ Copyright 2016, The authors, published by Springer Nature. d) Schematic rendering of flexible metasurface for electrically tunable perfect absorption at MIR region. e) The absorption spectra of the flexible metasurface absorber in the flat state with applied electrical current from 270 mA to 300 mA. f) The intensity distribution profiles of the magnetic field of a unit at an incident wavelength of 3.5 µm for insulating phase (top) and metallic phase (bottom) of VO_2_ film. Reproduced with permission.^[^ ^102^ ^]^ Copyright 2021, Wiley‐VCH. g) The unit cell diagram of the all‐dielectric metasurface with tunable resonant absorption at the NIR region. h) Wavelength and Si nanodisk diameter dependence of simulated maxima of electric dipole (ED) and magnetic dipole (MD) for the metasurface at insulating (40°) and metallic state (80°). i) Theoretically calculated and experimental absorption spectra for the metasurface at insulating (40°) and metallic state (80°) with Si nanodisk diameter of 510 nm (top two panels) and 550 nm (bottom two panels). Reproduced with permission.^[^ ^235^ ^]^ Copyright 2021, American Chemical Society.

Perfect absorbers possess the ability to absorb omnidirectional and unpolarized incident radiation over a certain waveband, showing extremely valuable applications in solar photovoltaics, photodetection, and photocatalysis.^[^ ^224^, ^225^, ^226^ ^]^ Perfect absorbers based on metasurfaces have been widely investigated for their superiorities of ultra‐thin thickness, easy integration, and excellent performance in light field modulation.^[^ ^227^, ^228^, ^229^, ^230^, ^231^ ^]^ Further, integrated with PCMs, metasurface absorbers with active tunability can be accomplished, which enable promising applications in integrated optoelectronic devices. Liu et al. reported a VO_2_‐based hybrid metasurface for electrically tunable perfect absorption (**Figure** **14**a).^[^ ^93^ ^]^ The metasurface adopts a typical sandwich structure with the metal‐dielectric‐metal (MDM) configuration, which is separated by Al_2_O_3_ spacer. The electrical current is applied through the top patterned Au layer to control the phase transition process. As clearly shown in Figure 14b, two resonant absorption modes, located at 3.05 µm and 3.9 µm, are supported in the metasurface absorber. The Joule heat generated in the top Au layer is transported to the underneath VO_2_ layer to induce IMT. The reflective spectra are continuously tuned with the increase of electrical current, which is ascribed to partial metallization and the accompanying change in permittivity of VO_2_. The modulation of the reflectance turns obvious when the current intensity exceeds ≈0.5 A and becomes saturated when it exceeds ≈1.0 A. By applying electrical pulses (4.0 A, 0.25 s), both the reflectance at resonant absorptions of 3.05 µm and 3.9 µm can be switched, demonstrating the dynamic modulation process (Figure 14c).

Flexible absorbers, which are deformable and can be bent, have potential applications in conformal optical devices, wearable photonics devices, and flexible cloaking.^[^ ^232^, ^233^, ^234^ ^]^ Recently, a flexible and electrically tunable metasurface absorber is proposed by Wang et al., as displayed in Figure 14d.^[^ ^102^ ^]^ Mica sheet is chosen as a flexible platform to support the upper layers. Similarly, the metasurface absorber has a standard MDM architecture with a graphene film on the top acting as the electrical heater. As shown in Figure 14e, for the flat state of the absorber, there is a distinct absorption peak with absorptivity close to 0.9 at the wavelength of ≈3.5 µm when VO_2_ is in the insulating phase. With the exciting current increasing, the IMT is triggered and the absorption peak gradually decreases due to the percolation progress. For the electrical current above 290 mA, the absorptivity decreases down to ≈0.2 at the whole measured MIR waveband, demonstrating a significant absorption modulation. For the bent state, the absorber has almost the same absorption spectra as the flat, indicating the flexible ability and incident angle‐independent merit. To investigate the deep mechanism, the magnetic field intensity distributions are given in Figure 14f. For the insulating phase of VO_2_, the magnetic field is concentrated inside the VO_2_, revealing a magnetic resonance. The localized enhanced electromagnetic field at the resonance causes great Ohmic loss, which is responsible for the near‐perfect absorption. Whereas there exists no magnetic resonance for the metallic phase of VO_2_ at 3.5 µm incidence, thus the absorption peak vanishes.

The MDM structure comprising metal and VO_2_ provides a general framework to realize electrically tunable metasurface absorbers. On the other hand, an all‐dielectric VO_2_‐based metasurface with tunable Mie‐resonance is also an optional solution to actively modulate optical absorption. Figure 14g shows the unit cell diagram of the all‐dielectric metasurface absorber designed by Tripathi et al.^[^ ^235^ ^]^ The Si nanodisks which support electric and magnetic dipole Mie resonances are adopted. The VO_2_ film acts as the absorption loss layer and provides active control by phase change. The electric and magnetic dipole modes overlap at the nanodisk diameter of 510 nm and separate with the diameter increasing (Figure 14h). Figure 14i shows consistent results between calculated and experimental results of the absorption spectra. For the absorber with the nanodisk diameter of 510 nm, two orders of magnitude amplitude modulation are achieved at a wavelength of 1370 nm, which is believed that the metasurface experiences a transformation from Huygen's metasurface to a coherent perfect absorption type. For the 550 nm one, the absorption peak shifts from 1450 nm (for the insulating state) to 1420 nm (for the metallic state), exhibiting a spectrally tunable perfect absorption.

## 5 NiTi‐Based Alloys

### 5.1 Tunable Terahertz Plasmonic Devices

**Figure 15:**
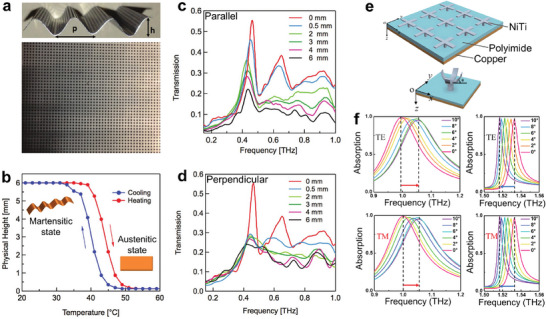
NiTi shape memory alloy for tunable terahertz plasmonic devices. a) Photographs of the patterned NiTi foils at curved (top) and flat (bottom) geometries. The *h* and *p* denote the height and period of the foil corrugation troughs. b) Measured thermal hysteresis in the phase change process of the NiTi foil and the two distinct shapes for the martensitic state and austenitic state. Measured transmission spectra of foils with various corrugation heights for incident light polarized **c)** parallel and d) perpendicular to the corrugation troughs. Reproduced with permission.^[^ ^240^ ^]^ Copyright 2017, Wiley‐VCH. e) Schematic of the proposed terahertz metasurface absorber. f) Numerically calculated absorption spectra with various bending angles (θ) of the cross for TE (top) and TM (bottom) polarized incidence. Reproduced under the terms of the CC‐BY Creative Commons Attribution 4.0 International license.^[^ ^241^ ^]^ Copyright 2021, The authors, published by Elsevier.

The Terahertz region (0.1 THz–10 THz) is a unique part of the electromagnetic spectrum lying between the infrared and microwave bands. Terahertz technology has shown critical application prospects in biological detection, wireless communication, medical imaging, and security checks.^[^ ^236^, ^237^, ^238^ ^]^ Consequently, it is important to develop terahertz devices such as terahertz sources, detectors, filters, switches, modulators, and absorbers. However, the weak interaction of terahertz waves with natural materials prevents the efficient manipulation. The development of metasurfaces provides an effective solution to manipulate terahertz waves for their tailored electromagnetic response.^[^ ^239^ ^]^ Gupta et al. fabricated a NiTi alloy‐based tunable plasmonic device for modulation of transmissive terahertz wave (**Figure** **15**a).^[^ ^240^ ^]^ The NiTi foil is patterned with a periodic array of subwavelength holes. The bistable physical geometries, curved and flat shapes, are formed after a shape memory training procedure, which can be switched to each other through thermal cycling with a hysteresis (Figure 15b). Figure 15c shows the transmission spectra of NiTi foil for incident light polarized to the NiTi foil corrugation troughs, where the resonance amplitudes decrease with the increase of corrugation height and this is associated with the increasing radiative losses of SPPs. The modulation of transmissive terahertz resonance is reversible through temperature control. For the perpendicular incidence, the resonances are suppressed for even small corrugation height (Figure 15d).

Terahertz absorbers have potential applications in the fields of electromagnetic cloaking, terahertz detectors, and sensors. Tunable terahertz absorbers are more attractive and are of great significance. A dynamically tunable terahertz metasurface absorber with a dual‐absorption band is proposed and theoretically analyzed (Figure 15e).^[^ ^241^ ^]^ The metasurface consists of the bottom copper film, middle polyimide film, and the top NiTi nanostructure arrays. The bending angle of the NiTi arms of the cross shape is characterized as θ, which is supposed to be controlled by thermal modulation. The calculated absorption spectra for the absorber with various bending angles are displayed in Figure 15f. The absorption peaks at low frequencies are on account of the excitation of magnetic dipole resonance, and the peaks at high frequencies are due to the resonance caused by the interactions of adjacent unit cells. With the increase of bending angle, for both TE and TM incidence, the resonance absorption peaks at low and high frequencies show a blueshift and a slight redshift, demonstrating tunability of the absorption peak in the cantilever bending progress.

### 5.2 Reconfigurable Bistable Optical Devices

**Figure 16:**
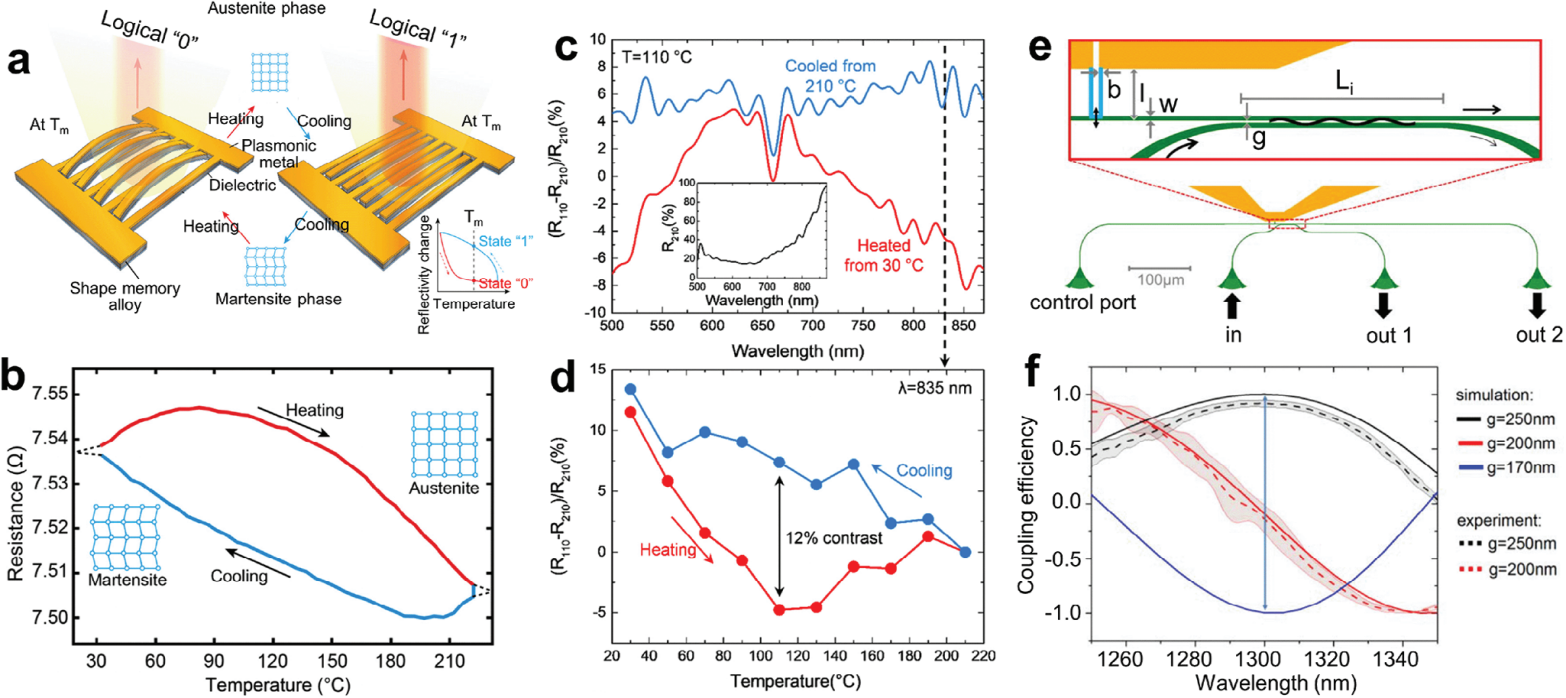
Reconfigurable bistable optical devices containing NiTi‐based alloy. a) Schematic rendering of the reconfigurable bistable nanostructure for rewritable memory. b) Hysteretic behavior of temperature‐dependent resistance of the 175 nm NiTi film deposited on a SiO_2_ substrate. c) Differential reflectivity spectra compared to the reference reflectivity at 210 °C after heating (red curve) and cooling to 110 °C. The inset shows the reflection spectrum at 210 °C. d) Hysteretic temperature‐dependence of reflection at the wavelength of 835 nm. Reproduced with permission.^[^ ^242^ ^]^ Copyright 2018, AIP Publishing. e) Architecture overview of NiTiCu optical waveguide device with two switchable output signal ports. The NiTiCu nanoactuator (blue), Si waveguide (green), and electrodes (yellow) are depicted in different colors. f) Simulated and experimental wavelength‐dependent coupling efficiency of the waveguide device for different gap sizes. The shaded areas indicate experimental standard deviation between different samples. Reproduced with permission.^[^ ^243^ ^]^ Copyright 2018, IEEE.

The shape memory effect supports optical memory behavior, which can be utilized to create reconfigurable bistable optical devices with optical memory or switch functions. Nagasaki et al. fabricated a shape memory nanograting device, which features two stable optical states under certain temperatures (**Figure** **16**a).^[^ ^242^ ^]^ The NiTi‐based nanograting device comprises suspended three layers of nanobeams, in which the NiTi film is deposited on a silicon nitride membrane and the Au film is on the top layer. The coated Au film can enhance the resonance optical response of the nanograting. For a given temperature, the nanograting has two different geometries due to the hysteresis effect during the thermal phase transformation between martensite and austenite, which correspond to two distinct optical responses and each of them denotes a stable state. The hysteretic effect in the phase change process can be visibly characterized through resistance measurement (Figure 16b). Figure 16c shows the differential reflectivity spectra at 110 °C for the heating and cooling parts of the hysteresis cycle. The reflectivity of the heated nanograting is higher than the cooled one over the whole measured waveband. The relative reflection contrast reaches 12% at 835 nm, distinguishing the two distinct states which denote logical “1” (high reflection) and logical “0” (low reflection), respectively (Figure 16d). The transition between the two states can be accomplished by thermal cycling, demonstrating the reconfigurable functionality.

NiTiCu, as a member of NiTi‐based SMAs, has been demonstrated to achieve optical waveguide devices with two switchable optical output ports (Figure 16e).^[^ ^243^ ^]^ The optical device is comprised of a NiTiCu bimorph nanoactuator and two Si waveguides. The nanoactuator adopts a double‐beam design for Joule heating‐driving mechanical force which originates from NiTiCu phase transition. The two waveguides have a freestanding coupling junction with a narrow gap *g*, which can be reversibly tuned by the nanoactuator through cantilever deflection and thus modulate evanescent wave coupling. Figure 16f shows the wavelength‐dependent coupling efficiency for different gap sizes. At the wavelength of 1300 nm, with the gap width decreasing from 250 to 200 nm stimulated by NiTiCu phase transition, the experimental coupling efficiency changes from 0.9 to −0.1, corresponding to a 53% reduction in power transfer to output port 2. Notably, the simulation result further suggests that a 100 nm gap would cause a 100% power transfer reduction (coupling efficiency of −1) to port 2, namely complete light output from port 1. Moreover, the cantilever deflection was demonstrated to be precisely reproducible by controlling the heating power.

## 6 Summary and Outlook

In this review, a series of PCMs (GST, VO_2_, NiTi‐based alloys, GSST, AIST, Sb_2_S_3_, and GeTe) belonging to different material categories and their respective functional RMNODs are systematically introduced. PCMs provide an extremely fascinating pathway to achieve reversible modulation in MNODs. Remarkable progress has been made in the design and fabrication of RMNODs in recent years. The representative achievements discussed in this review are listed in **Table** **1** based on their optical functions and the containing PCMs. GST is one of the most well‐known chalcogenide PCMs to achieve reconfigurable metasurfaces, reconfigurable on‐chip photonic devices, and tunable optical film devices. Meanwhile, the above devices based on Sb_2_S_3_ and GSST have also been investigated due to their broad transparency in VIS and NIR regions. The plasmonic‐dielectric transitions of VO_2_ with ultrafast transition speed make it suitable for developing photodetectors, all‐optical switches, and tunable metasurface absorbers. The volatile phase change attribute of VO_2_ makes it much easier to realize the reconfigurable process. NiTi‐based SMAs, leveraging the shape memory effect, are employed in developing tunable terahertz plasmonic devices and reconfigurable bistable optical devices, offering significant potential for advancement. The working wavebands of RMNODs cover terahertz to UV regions, indicating tailored optical responses within broadband. There are various excitation methods (thermal, optical, electrical, mechanical, electronic, magnetic, and electrochemical) to induce phase transition in RMNODs, among which electrical and optical stimulation ways are more convenient, controllable, valuable, and more worthy of being adopted in future applications. The RMNODs take full advantage of the significant contrasts in material characteristics (permittivity or shape) induced by phase transitions, presenting the distinction in the form of optical responses by virtue of carefully designed micro/nano‐structures. More specifically, the amplitude, phase, and resonance of incident light as well as the photoelectric response can be reversibly tuned in active or passive manners, and thus the desirable optical functions are realized. These artificial RMNODs provide strong technical support to the next‐generation photonic systems with high levels of integration, responsiveness, endurance, and efficiency in respect of optical switch, photonic memory, light transmission, optical signal modulation, nanoscale color display, and photoelectric conversion.

**Table 1:** Summary of the RMNODs based on PCMs.

Optical functions	PCMs	Deposition methods	PCM shapes	Device structures	Working wavebands	Excitations of phase change	Ref.
Reconfigurable metasurfaces	GSST	Thermal evaporation	Disk	Al_2_O_3_/GST/Al_2_O_3_/Pt	1550 nm	Electrical pulses	[58]
	GST	Magnetron sputtering	Strip	Al_2_O_3_/GST/Al_2_O_3_/Ag	VIS	Electrical pulses	[57]
			Film	Au/Al_2_O_3_/GST/ Al_2_O_3_/Au/Al_2_O_3_/W/HfO_2_	NIR	Electrical pulses	[94]
			Film	ZnS‐SiO_2_/GST/ZnS‐SiO_2_/Au	NIR	Laser pulses	[59]
			Film	ZnS‐SiO_2_/GST/ZnS‐SiO_2_	NIR	Laser pulses	[60]
			Strip	ZnS‐SiO_2_/GST/ZnS‐SiO_2_	UV‐VIS	Laser pulses	[157]
			Film	stacked GST/Au	NIR	Heating/laser pulses	[161]
	Sb_2_S_3_	Magnetron sputtering	Patterned film	PMMA/Sb_2_S_3_/ITO/	VIS	Heating/laser pulses	[160]
Reconfigurable on‐chip photonic devices	GST	Magnetron sputtering	Strip	Al_2_O_3_/GST/Si	NIR	Electrical pulses	[100]
			Strip	SiO_2_/GST/Au nanogap/Si_3_N_4_	NIR	Electrical pulses	[92]
			Strip	Al_2_O_3_/GST/Al_2_O_3_/ SLG/Au/Ti/Al_2_O_3_/Si	NIR	Electrical pulses	[172]
			Strip	ITO/GST/Si_3_N_4_	NIR	Laser pulses	[106]
			Slice	Si_3_N_4_/GST/SiO_2_/Si	NIR	Laser pulses	[166]
			Slice	ITO/GST/Si_3_N_4_	NIR	Laser pulses	[178]
	Sb_2_S_3_	Magnetron sputtering	Slice	ITO/Sb_2_S_3_/Si/SiO_2_	NIR	Electrical pulses	[173]
	GSST	Thermal evaporation	Strip	SiO_2_/GSST/SiN	NIR	Laser pulses	[108]
Tunable optical film devices	GST	Magnetron sputtering	Film	ITO/GST/ITO/Pt	VIS	Electrical pulses	[188]
	AIST	Magnetron sputtering	Film	ITO/AIST/ITO/Pt	VIS	Electrical pulses	[191]
	GeTe	Magnetron sputtering	Film	MgF_2_/TiO_2_/GeTe/ TiO_2_/GeTe/SiO_2_/ Pd/NiCr	VIS	Electrical pulses	[104]
	Sb_2_S_3_	Magnetron sputtering	Film	Si_3_N_4_/Sb_2_S_3_/Si_3_N_4_/Al	VIS	Heating/laser pulses	[105]
	GST/ Sb_2_S_3_	Magnetron sputtering	Film	GST/Ag/Sb_2_S_3_/W	VIS	Electrical current	[198]
	Sb_2_S_3_	Magnetron sputtering	Film	Ge/Ag/Sb_2_S_3_/Ag	VIS	Electrical current	[199]
VO_2_‐based Photodetectors	VO_2_	Magnetron sputtering/annealing	Film	Au/VO_2_	NIR	Laser	[216]
		Magnetron sputtering/annealing	Film	WSe_2_/VO_2_/SiO_2_	VIS	Heating	[219]
		Pulsed laser deposition	Film	Transistor	UV	UV irradiation/Electrolyte gating	[156]
All‐optical switches	VO_2_	Atomic layer deposition	Conformal coating	VO_2_/ITO nanorod array	NIR	Laser pulses	[61]
		‐	Film	Au/VO_2_	THz	THz pulses	[222]
		‐	Patterned film	VO_2_/substrate	THz	THz pulses	[223]
Tunable metasurface absorbers	VO_2_	Reactive ion sputtering	Film	Au/Al_2_O_3_/VO_2_/Al_2_O_3_/Au	MIR	Electrical current/electrical pulses	[93]
		Electron beam evaporation	Film	Graphene/Au/VO_2_/Au/Mica	MIR	Electrical current	[102]
		Magnetron sputtering	Film	PMMA/Si/SiO_2_/VO_2_	NIR	Heating	[235]
Tunable terahertz plasmonic devices	NiTi	‐	Patterned foil	NiTi patterned foil	THz	Heating	[240]
		‐	Cross	NiTi/polyimide/Cu	THz	Heating	[241]
Reconfigurable bistable optical devices	NiTi	Magnetron sputtering	Strip	Au/NiTi/SiN	VIS‐NIR	Heating	[242]
	NiTiCu	Magnetron sputtering	Cantilever	NiTiCu/Si	NIR	Heating	[243]

Although significant achievements have been made in the development process of RMNODs, some issues that limit the practical applications still exist and remain to be resolved. First, Joule heat generated within the electrically and optically driven phase transition processes would lead to the heating problem, which is inconsequential in a single optical device but nonnegligible in the integrated system. Second, high amorphization temperatures of GST indicate high energy consumption where sufficient energy is required to reach its melting point. Cyclical conversion between the crystalline and amorphous states of GST would lead to high power consumption. Third, inhomogeneous temperature distribution in metal heaters would cause uniformity of phase transition of PCMs, which is also a critical challenge.^[^ ^58^ ^]^ Fourth, the inevitable dielectric loss of light would decrease transmission efficiency, and this circumstance is more severe in plasmonic structure‐contained optical devices due to inevitable reflection and absorption. Finally, the structural shapes of MNODs largely determine the electromagnetic mode (e.g., SPPs, LSPR, electric dipole resonance, magnetic dipole resonance). Reasonable structure configuration could greatly improve the optical performance. However, different from the universal transistor configurations in electrical devices, MNODs can be constituted by components with various kinds of structures, such as multilayer, split resonant ring, waveguide, nanorod, nanocone, and optical grating. So far, the result‐oriented forward numerical simulation is the mainstream scheme in optical structure design, which is experience‐dependent, time‐consuming, and inefficient in theoretically constructing micro/nano‐optical structures.

In conclusion, developing functional RMNODs to build photonic systems with high integration density, fast responsiveness, remarkable endurance, high efficiency, and lower power consumption is of great realistic necessity. On the one hand, solving the above problems is of great significance for promoting the practical application process of RMNODs. On the other hand, enhancing the working performance of RMNODs is crucial, as it significantly influences their practicability. For the PCM‐based reconfigurable metasurfaces operating in the VIS‐NIR region, state transition usually occurs throughout the entire PCM. While pixel‐by‐pixel control of the structure units can significantly enhance the modulation flexibility and increase transmission channels, enabling superior wavefront regulation ability.^[^ ^244^ ^]^ Besides, electrical tuning of RMNODs is favored due to its compact volume and convenient phase change control. Adopting the CMOS‐compatible PIN diode heating scheme to achieve multi‐level light intensity manipulation could significantly improve integration level, reduce power dissipation, and boost information storage capacity.

In the future, in addition to further investigate RMNODs based on existing PCMs, other PCMs (e.g., MoX_2_ (X = S, Se, Te),^[^ ^245^, ^246^, ^247^ ^]^ organic PCMs,^[^ ^248^, ^249^ ^]^ cesium/formamidinium lead iodide perovskites^[^ ^250^, ^251^ ^]^) can also be considered. Selecting PCMs with relatively low phase change temperatures is a feasible strategy to reduce the power consumption of driving phase transition. Doping is a common method to improve the physical properties of materials, which can be considered for PCMs. Moreover, the coupling of different materials is expected to broaden the modulation scope of RMNODs. For instance, combining PCMs with 2D materials to constitute hybrid systems may enrich the functionality of RMNODs, pairing GST with VO_2_ would enhance the modulation degree of freedom, and coupling PCMs with nonlinear optical materials (e.g., LiNbO_3_) would lead to reconfigurable nonlinear optical modulation. Besides, it is advisable to adopt electrostatic force in the shape memory training procedures of SMA micro/nanostructures.^[^ ^45^ ^]^ With the development of artificial intelligence, machine learning, as an emerging approach that deduces inherent rules and optimizes performance utilizing specific algorithms with the use of given data, has been applied in designing metasurfaces and yields some meaningful results.^[^ ^252^, ^253^, ^254^ ^]^ Consequently, it is advisable to adopt a machine learning approach in the task of predicting complicated RMNOD structures.

## Conflict of Interest

The authors declare no conflict of interest.
